# The Role of Artificial Intelligence in Advancing Biosensor Technology: Past, Present, and Future Perspectives

**DOI:** 10.1002/adma.202504796

**Published:** 2025-06-16

**Authors:** Tuğba Akkaş, Mahshid Reshadsedghi, Mustafa Şen, Volkan Kılıç, Nesrin Horzum

**Affiliations:** ^1^ Department of Biomedical Engineering Graduate Program Izmir Katip Celebi University Izmir 35620 Turkey; ^2^ Department of Electrical and Electronics Engineering Graduate Program Izmir Katip Celebi University Izmir 35620 Turkey; ^3^ Department of Engineering Science Izmir Katip Celebi University Izmir 35620 Turkey

**Keywords:** artificial intelligence, biosensors, Internet of Things (IoT), wearable devices

## Abstract

Integrating artificial intelligence (AI) into biosensor technology enables data processing, quantitative analysis, real‐time decision‐making, and adaptive sensing capabilities through advanced pattern recognition and predictive modeling. In addition, AI has the potential to drive innovation in the design of advanced materials for biosensing applications by reducing the reliance on trial‐and‐error methods. This review explores the transformative impact of AI on biosensor technology in the context of historical development, current status, and future prospects. It begins with an overview of the evolution of AI, biosensor technology, and their integration. Comparative analysis of AI‐driven innovations in optical, fluorometric, and electrochemical biosensors is presented, highlighting how AI can improve sensor performance. The role of advanced materials on the development of AI‐assisted biosensors is also discussed as the choice of material has a profound effect on biosensor capabilities. Applications of AI‐assisted biosensors are comprehensively explored across healthcare, environmental monitoring, food safety, and agriculture. This study concludes by addressing challenges, opportunities, ethical concerns, and future research directions, providing a comprehensive and up‐to‐date resource for researchers.

## Graphical abstract

This review explores the transformative role of AI in biosensor technology and provides a holistic interdisciplinary perspective that covers a broader scope of AI‐enabled biosensor technologies across various sectors including healthcare, environmental monitoring, food safety, and agriculture. It also highlights the important role of novel materials in advancing AI‐assisted biosensors in terms of sensitivity, specificity, and data quality.

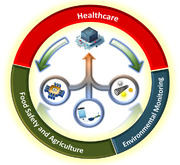

## 1 Introduction

A biosensor is an analytical device that combines a biorecognition element, typically derived from a living organism, with a physicochemical transducer that translates the biorecognition event into a quantifiable signal. Since their introduction, biosensors have evolved significantly, addressing challenges in diverse fields such as medicine, environmental monitoring, and beyond.^[^ ^1^ ^]^ The development of new biorecognition elements, such as antibodies and aptamers, together with advanced detection methods like field‐effect transistors and quartz crystal microbalance, has further broadened the potential and capabilities of biosensors.^[^ ^2^ ^]^ They are now seamlessly integrated into smartphones and wearable monitoring systems, as well as lab‐on‐chip and organ‐on‐chip technologies.^[^ ^3^ ^]^ Concurrently, the growing focus on molecular biology has led to the development of affinity biosensors capable of detecting very low levels of DNA and proteins, with broad applications in biomedicine.^[^ ^4^, ^5^ ^]^ The introduction of surface plasmon resonance (SPR) technology has made significant contributions to various fields such as biotechnology and drug development in terms of real‐time and label‐free monitoring of biomolecular interactions.^[^ ^6^ ^]^ The integration of advanced nanomaterials including carbon nanotubes (CNTs), graphene, metal nanoparticles (NPs), and metal‐organic frameworks (MOFs) has transformed biosensor technology, by enhancing sensitivity, selectivity, functionality, and miniaturization.^[^ ^7^, ^8^ ^]^ The development of point‐of‐care testing devices and wearable biosensors has attracted an increasing amount of attention in the last decade due to their potential applications in real‐time health monitoring and early disease diagnosis.^[^ ^9^ ^]^ Although glucose biosensors dominate the current market due to high demand, there is a growing interest in designing biosensors to detect diverse analytes in fields such as food safety, agriculture, drug discovery, and medical diagnostics.^[^ ^10^, ^11^, ^12^ ^]^

**Figure 1:**
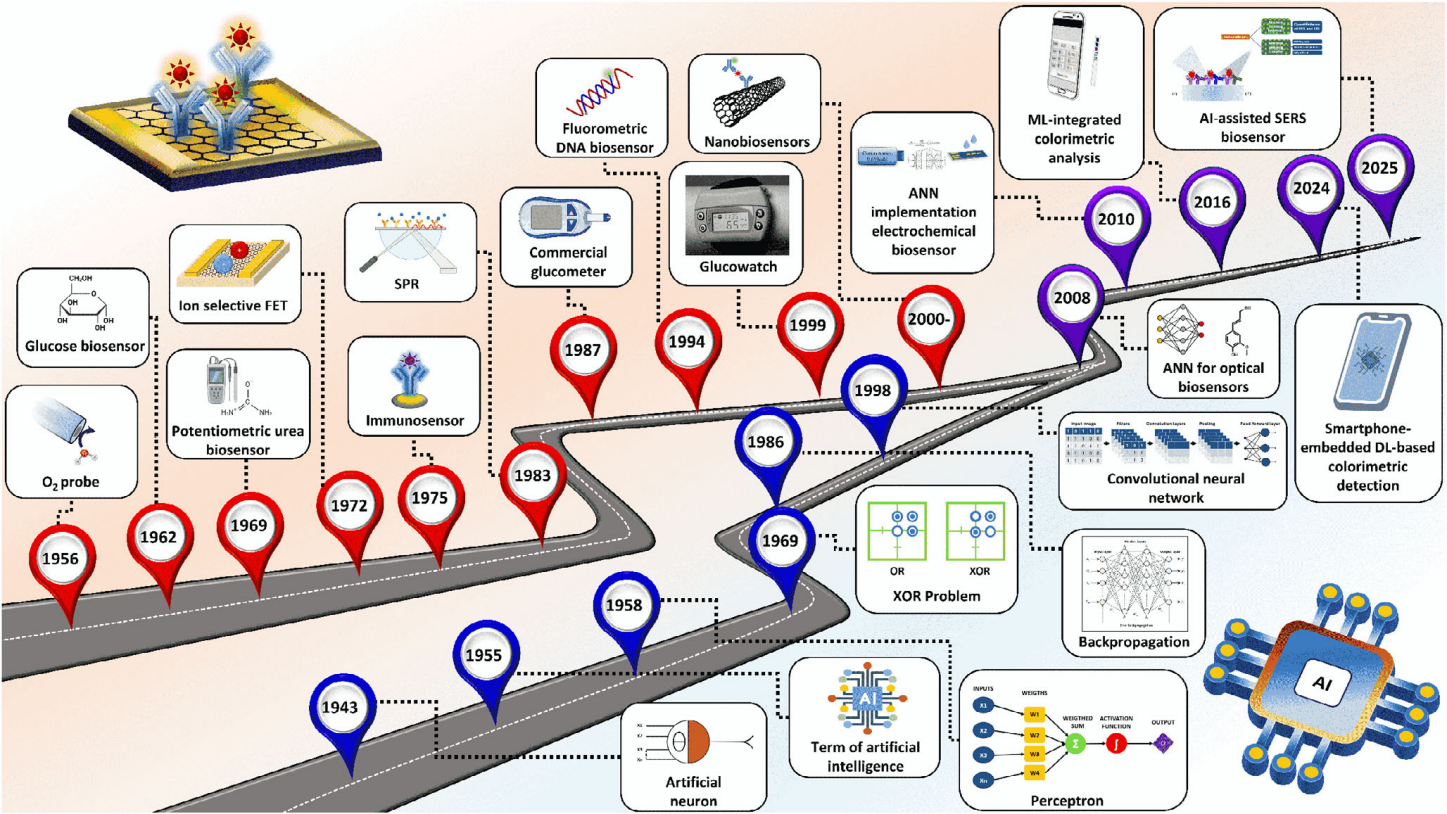
Timeline of biosensor and AI development and their intersections. “GlucoWatch” Reproduced with permission,^[^ ^161^ ^]^ Copyright 2001, Elsevier. This figure was created using icons from Freepik (www.freepik.com).

Advances in algorithms, hardware, and computing power have significantly impacted the widespread implementation of biosensors.^[^ ^13^ ^]^ New trends focus on using intelligent and interconnected systems that integrate big data analytics, the Internet of Things (IoT), and AI.^[^ ^14^ ^]^ AI has evolved from early concepts introduced by pioneers such as Alan Turing in the mid‐20th century,^[^ ^15^ ^]^ to expert systems in the 1980s,^[^ ^16^ ^]^ and to advanced machine learning (ML) and deep learning (DL) algorithms today (**Figure** **1**). Advanced DL and ML models can drastically enhance real‐time predictive accuracy of biosensors through fast processing of large datasets, effectively identifying complex patterns, weak trends, and anomalies.^[^ ^17^, ^18^, ^19^, ^20^ ^]^ AI‐integrated systems hold great potential to be instrumental for the development of adaptive monitoring platforms capable of making real‐time decisions in dynamically changing conditions.^[^ ^21^ ^]^ Besides, AI can significantly enhance the sensitivity, specificity, and stability of biosensors by filtering out undesirable noise and signals to provide more accurate and reliable measurements.^[^ ^22^, ^23^, ^24^ ^]^ Considering the rapid progress of AI‐assisted biosensors across various disciplines, a comprehensive review of current developments can serve as a valuable reference for researchers. The integration of AI into biosensor technology has been reviewed in several papers published within the last two years.^[^ ^20^, ^25^, ^26^ ^]^ Most of these reviews focus on a specific aspect of AI‐assisted biosensors in healthcare. For instance, Zhang et al. reviewed recent papers on AI‐assisted wearable biosensing devices in disease diagnostics and fatigue, focusing on personalized medicine.^[^ ^22^ ^]^ Similarly, Bhaiyya et al. examined the role of ML‐assisted biosensors in Point‐of‐Care‐Testing to assist clinical decision processes.^[^ ^21^ ^]^ Ramalingam et al. discussed the impact of nanotechnology on both traditional and AI‐assisted biosensor strategies, specifically for virus detection.^[^ ^27^ ^]^ These reviews, although valuable, do not provide a holistic interdisciplinary perspective that covers a broader scope of AI‐assisted biosensor technologies across various sectors. This paper presents an overview of research articles published since 2020, offering an assessment of state‐of‐the‐art technologies, methodologies, and applications at the intersection of AI and biosensor technology, while also exploring the role of material science. After a brief overview of ML and DL algorithms, their implementation to the analysis of biosensor data is discussed, and the advantages of AI in real‐time applications are highlighted. Along with progress in terms of sensing methodologies and integration of advanced materials, the applications of AI‐assisted biosensors in healthcare, environmental monitoring, food safety, and agriculture are comprehensively explored. Finally, the paper concludes with an analysis of the challenges, opportunities, and future directions.

## 2 Ai‐Enhanced Biosensor Technology: Evolution and State‐of‐the‐Art

### 2.1 AI in Data Processing and Interpretation

Implementing AI algorithms has become instrumental in biosensor data analysis, as their computational architectures are uniquely suited to process and extract statistically significant patterns from complex, high‐dimensional biological datasets. Biosensor‐derived data in its raw form requires systematic pre‐processing steps to attenuate signal noise and optimize signal quality, facilitating robust analytical interpretation. In this regard, AI offers comprehensive analytical capabilities for both pre‐processing biological signals to improve signal quality and post‐processing to interpret data, extract meaningful patterns, and support real‐time decision‐making.^[^ ^28^, ^29^ ^]^

ML is a subset of AI involving algorithms that enable computer systems to learn from data and make decisions without requiring explicit task‐specific programming. ML algorithms can be categorized into four types based on their learning approach: supervised learning, unsupervised learning, semi‐supervised learning, and reinforcement learning (RL). In supervised learning, models are trained on labeled data, which is carefully annotated with meaningful tags that classify the elements of data or outcomes, allowing the system to learn patterns and make accurate predictions.^[^ ^30^ ^]^ In contrast, unsupervised learning works with unlabeled data, where the system receives only the input data without any corresponding output labels, aiming to uncover hidden structures and relationships within the dataset.^[^ ^31^ ^]^ Semi‐supervised learning combines elements of both supervised and unsupervised learning by utilizing a small amount of labeled data alongside a large pool of unlabeled data to improve model learning and decision‐making. It is particularly useful when unlabeled data is abundant and labeled data is scarce and expensive to obtain.^[^ ^32^ ^]^ RL employs an intelligent agent to interact with a dynamic environment to maximize a reward signal. Unlike supervised learning, RL does not rely on labeled input‐output pairs or explicit corrections for sub‐optimal actions. Instead, it is an online learning process in which the agent learns through a trial and error procedure, taking actions, and receiving feedback in the form of rewards.^[^ ^33^ ^]^

Classification and regression are two key tasks in supervised learning. Classification involves assigning input data to predefined categories or classes, such as identifying whether a biosensor reading indicates a healthy or diseased state.^[^ ^34^ ^]^ Regression, on the other hand, predicts quantitative values, such as estimating the concentration of a biomarker from sensor data.^[^ ^35^ ^]^ Among the many ML algorithms, support vector machines (SVM) are a powerful supervised learning technique widely used for classification and regression tasks.^[^ ^36^ ^]^ SVM operates by mapping input data into a higher‐dimensional space using a kernel function and then identifying the optimal hyperplane that separates data points from different classes.^[^ ^37^ ^]^ This hyperplane is defined by maximizing the margin, the distance between the nearest data points (support vectors) of each class, and the hyperplane. SVMs are especially effective for datasets where the classes are not linearly separable, as kernel functions like the radial basis function or polynomial kernels enable non‐linear decision boundaries.^[^ ^38^ ^]^ Random forests (RF), on the other hand, are an ensemble learning method that constructs multiple decision trees (DTs) during training and aggregates their outputs to improve prediction accuracy and robustness.^[^ ^39^ ^]^ Each DT in an RF is trained on a randomly sampled subset of the data (with replacement) and a random subset of features, a process known as bootstrap aggregating or bagging. This approach helps reduce the risk of overfitting, a situation that occurs when a model makes accurate predictions on training data but fails to generalize to testing data. By averaging or voting across multiple trees, RF improves generalization, ensuring better performance on unseen data.^[^ ^40^, ^41^ ^]^ The k‐nearest neighbors (k‐NN) algorithm is a nonparametric, supervised learning method employed for both classification and regression. In classification, it assigns a class to a data point based on the majority label of its nearest neighbors in the feature space. The number of neighbors (k) is a critical hyperparameter that determines the behavior of the model k values, resulting in more sensitive classifications, while larger k values produce smoother decision boundaries. For regression, k‐NN predicts a value by calculating the mean or median of the target variable among the k‐most similar instances.^[^ ^42^ ^]^ Despite its simplicity, k‐NN performs well in scenarios where the decision boundaries are irregular or complex. However, the method is computationally intensive for large datasets, as it requires calculating the distance between the target point and all points in the training set during inference.^[^ ^30^ ^]^

DL, on the other hand, is a specialized subset of ML based on neural network architectures, designed to automatically learn hierarchical representations of data, making it particularly effective for tasks involving large‐scale and complex datasets, such as medical image recognition and EEG signal processing.^[^ ^24^ ^]^ Unlike traditional ML algorithms, which typically require manual feature extraction before classification or regression, DL integrates feature extraction through multi‐layered neural networks that automatically learn hierarchical representations from raw data.^[^ ^43^ ^]^ Through successive layers, DL algorithms initially capture low‐level features, such as edges or textures in images, and progressively build higher‐level abstractions, such as shapes or objects, without the need for explicit human intervention.^[^ ^44^ ^]^ DL algorithms, particularly convolutional neural networks (CNNs) and recurrent neural networks (RNNs) have revolutionized numerous domains by enabling high‐level reasoning, such as identifying early‐stage cancer in medical images like MRIs and X‐ray,^[^ ^45^ ^]^ detecting environmental risks or pathogens using smart sensors^[^ ^46^ ^]^ and sophisticated feature extraction.^[^ ^47^ ^]^ Over the last decade, several CNN architectures have been developed to enhance performance in various applications,^[^ ^48^ ^]^ including disease detection through wearable sensors,^[^ ^49^ ^]^ brain activity analysis using EEG data,^[^ ^50^ ^]^ and continuous glucose monitoring for diabetes.^[^ ^51^ ^]^ While traditional 2D CNNs have demonstrated considerable success in processing image‐based biosensor data, emerging architectures such as 3D CNNs, Variational Autoencoders (VAEs), Transformers, and deep image priors have expanded the analytical capabilities of AI in biosensing. 3D CNNs have emerged as a powerful extension of conventional 2D CNNs, particularly well‐suited for spatiotemporal data analysis. Unlike 2D CNNs, which extract spatial features from individual frames or slices, 3D CNNs operate across three dimensions: height, width, and depth (or time),^[^ ^52^ ^]^ making them ideal for processing volumetric medical data such as CT or MRI scans,^[^ ^53^ ^]^ as well as continuous biosensor streams like video analysis or real‐time monitoring signals.^[^ ^54^ ^]^ In biosensing contexts, 3D CNNs can model complex temporal‐spatial dependencies, like tracking metabolic changes over time.^[^ ^55^ ^]^ This capability enables more accurate and context‐aware classifications, such as differentiating abnormalities across various organs.^[^ ^56^ ^]^

Variational Autoencoders (VAEs) are a class of generative models that offer probabilistic interpretations of data through latent variable modeling. VAEs have proven highly effective in denoising, dimensionality reduction, and generating synthetic biosensor data for model training augmentation particularly when labeled datasets are limited.^[^ ^57^ ^]^ In biosensor applications, VAEs can reconstruct input signals from compressed representations, allowing for the suppression of noise while preserving relevant features. This makes them invaluable in preprocessing steps where signal quality is compromised, such as low‐quality EEG or electrocardiogram (ECG) signals.^[^ ^58^ ^]^ Moreover, VAEs facilitate anomaly detection by modeling the normal distribution of biosensor outputs and identifying deviations that may signal pathological conditions.^[^ ^59^ ^]^ Transformers, unlike CNNs and RNNs which rely on convolutional or sequential operations, utilize self‐attention mechanisms to capture global dependencies across the input data, regardless of position or sequence.^[^ ^60^ ^]^ Vision Transformers (ViTs), for instance, have demonstrated strong performance in image classification tasks by segmenting input images into patches and processing them through layers of multi‐head attention and feed‐forward networks.^[^ ^61^ ^]^ In biosensors, transformer‐based models have shown promise in analyzing multichannel time‐series data, such as multi‐lead ECG or EEG, where long‐range dependencies and cross‐channel interactions play a critical role in diagnosis. Since their initial application to ECG analysis in 2022,^[^ ^62^ ^]^ transformers have marked a significant advancement over traditional RNNs, primarily owing to their self‐attention mechanisms and ability to process entire input sequences in parallel. This architectural advantage not only improves computational efficiency but also facilitates real‐time signal monitoring.^[^ ^63^, ^64^ ^]^ Deep image priors represent another innovative direction in AI‐enhanced biosensor data analysis. Unlike conventional supervised DL models, deep image priors exploit the implicit structure of CNNs to perform image restoration tasks such as denoising, inpainting, or super‐resolution without requiring external training data.^[^ ^65^ ^]^ This approach is particularly beneficial in biosensor imaging scenarios with limited annotated data or where signal degradation is common. The integration of advanced architectures has significantly improved the performance and flexibility of AI in biosensor applications. These approaches overcome the limitations of traditional CNNs and RNNs, offering more scalable and efficient solutions for real‐time accurate biosensor analysis.

**Figure 2:**
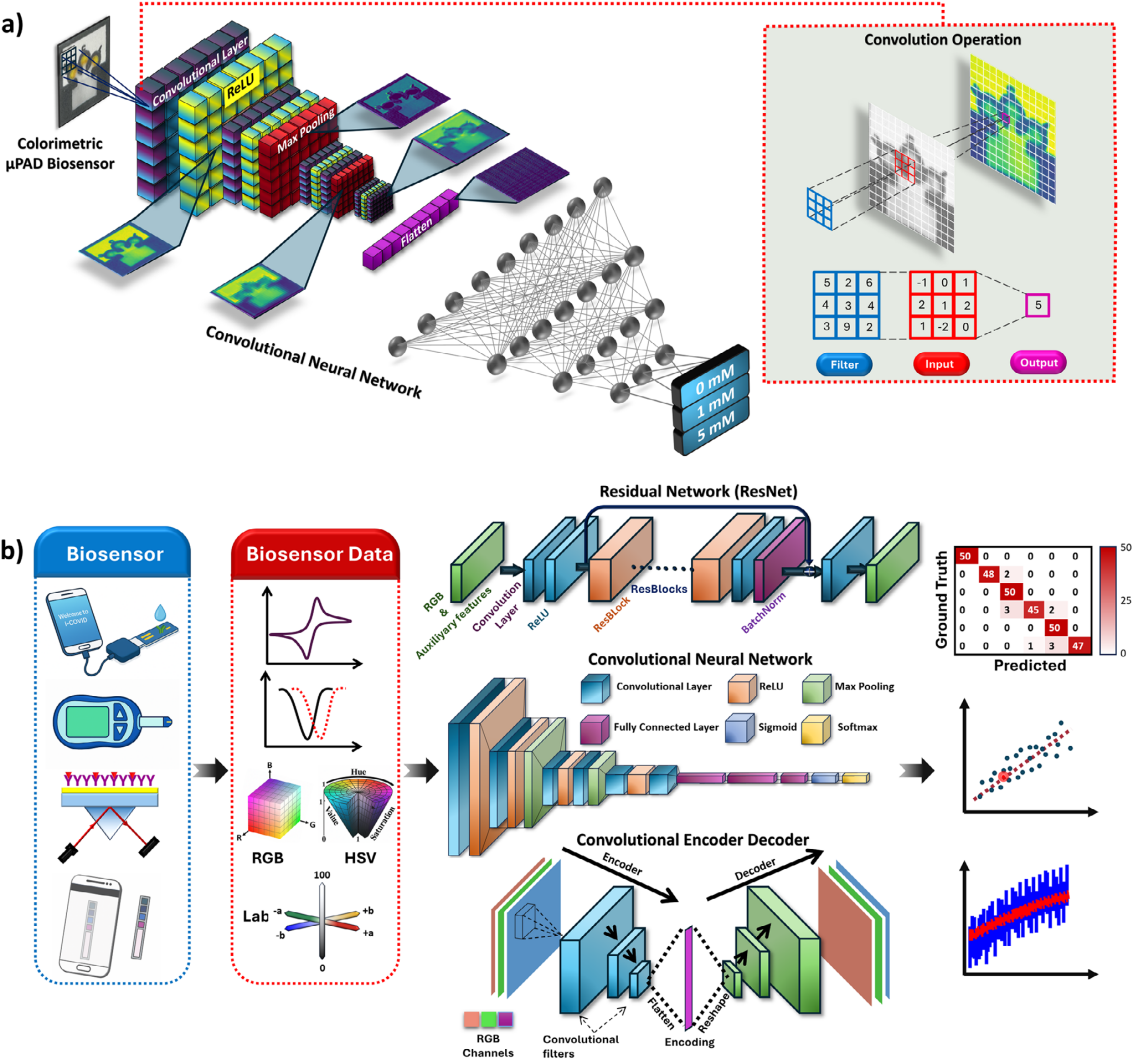
A layer‐wise demonstration of feature extraction and classification in a CNN architecture, with convolution operations (inset) used for a colorimetric biosensor (a). An illustration depicting the workflow of applying various AI networks to different biosensor types and data modalities for classification, regression, and noise reduction (b).

In parallel, developments including structural enhancements, advanced regularization techniques, and optimized training methods have significantly improved artificial neural networks (ANNs), particularly CNNs, and RNNs, resulting in improved efficiency, scalability, and accuracy.^[^ ^66^ ^]^ The architectural paradigm of ANNs, the backbone of many modern AI systems, is fundamentally inspired by the information processing patterns exhibited in biological neuronal networks.^[^ ^67^ ^]^ CNNs, a specialized type of ANN, achieved widespread recognition following the introduction of AlexNet in 2012.^[^ ^68^ ^]^ CNNs are particularly well‐suited for processing image‐based data, leveraging their layered architecture to extract and analyze complex features such as edges, textures, shapes, and objects.^[^ ^69^, ^70^ ^]^ The process begins with an input image, which serves as raw data for the network, containing spatial patterns and structures that the CNN learns to recognize.^[^ ^71^, ^72^ ^]^ The first transformation occurs in the convolutional layer, where the image passes through filters (kernels) designed to detect essential patterns. The output consists of multiple feature maps, each highlighting different aspects of the original image.^[^ ^73^, ^74^ ^]^ The rectified linear unit (ReLU) activation function is applied to improve the ability to learn complex patterns. It sets negative values to zero while allowing positive inputs to pass unchanged, effectively introducing non‐linearity without compromising computational efficiency.^[^ ^75^ ^]^ Next, max‐pooling reduces the spatial dimensions of the feature maps while preserving critical features, improving computational efficiency, and preventing overfitting.^[^ ^76^ ^]^ The extracted features are then flattened into a one‐dimensional vector, preparing them for the next stage, fully connected layers. In the fully connected neural network, these transformed features are processed through multiple layers of neurons, enabling high‐level reasoning and decision‐making.^[^ ^77^ ^]^ Finally, the network produces an output, such as classification probabilities or specific predictions, ensuring meaningful pattern extraction for accurate results^[^ ^78^ ^]^ (**Figure** **2**a). Additionally, CNNs leverage a hierarchical feature learning process, allowing them to progressively extract different levels of abstraction from an image. Initially, at the low‐level feature stage, the network detects basic elements such as edges and simple textures. As the image moves through deeper layers, mid‐level features emerge, capturing patterns like corners and contours. Finally, at the high‐level feature stage, the network learns more abstract representations, such as shapes and structures, enabling accurate classification and recognition. This structured approach to feature extraction ensures that CNNs can effectively understand and analyze complex image structures.^[^ ^48^, ^79^ ^]^ Recently, architectural improvements such as skip connections in ResNet^[^ ^80^ ^]^ and the encoder‐decoder structure in Convolutional Encoder‐Decoder networks have further enhanced the capabilities of CNNs, particularly for tasks involving classification, regression, and noise reduction across diverse biosensor data modalities (Figure 2b).

Concurrently, RNNs have been developed to process sequential data, offering a distinct advantage in tasks that depend on understanding context and temporal relationships. Unlike CNNs, RNNs employ feedback loops that allow information to persist over time steps, which makes them particularly well suited for applications such as continuous monitoring of heart rate,^[^ ^81^ ^]^ blood glucose levels,^[^ ^82^ ^]^ and the detection of abnormalities in physiological signals over time.^[^ ^83^ ^]^ However, conventional RNNs have difficulties due to vanishing and exploding gradient problems, which can restrict their ability to learn effectively over long sequences. These problems are addressed by Long Short‐Term Memory (LSTM) units, which are designed to retain essential information even over extended time steps (long‐term memory), while still tending to prioritize more recent information (short‐term memory). However, LSTMs may still struggle to retain critical details that are distant from the current point of analysis. To address this limitation, Gated Recurrent Units (GRU) were introduced, utilizing update and reset gates to regulate the flow of information. This enables GRUs to retain important information without the risk of gradient decay over time, allowing more effective modeling of long‐range dependencies and sequential data.^[^ ^84^, ^85^ ^]^ These improvements have strengthened the ability of RNNs to capture long‐term dependencies, enhancing their performance on complex tasks such as text generation and language translation. CNNs and RNNs collectively illustrate the expanding capabilities of ANNs in addressing challenges, often in complementary ways. CNNs outperform at capturing spatial hierarchies in image data, while RNNs are especially effective at modeling sequential patterns.^[^ ^86^ ^]^ However, the complexity of causality in biological systems presents significant challenges in accurately interpreting large amounts of data. Biological processes are influenced by many interconnected factors, such as genetics, environment, and epigenetics, which can create non‐linear and dynamic relationships.^[^ ^87^ ^]^ Furthermore, biological data are often noisy or incomplete, and the large volume of data from technologies such as genomic sequencing complicates the extraction of clear patterns.^[^ ^88^ ^]^ To overcome these challenges, AI has emerged as a transformative solution that significantly enhances the field of biosensors. AI, especially through the application of ML and DL algorithms, improves the ability of biosensors to process complex biological signals and extract meaningful insights from large datasets.^[^ ^89^ ^]^

### 2.2 Advances Based on Detection Methods

#### 2.2.1 Optical Detection

An optical biosensor converts optical changes ‐such as absorbance, fluorescence, photon scattering, refractive index changes, SPR, and color‐ resulting from a biorecognition event into an electrical signal that can be processed, amplified, and displayed.^[^ ^90^ ^]^ Among various optical detection methods, colorimetric detection has attracted considerable attention as an analytical tool designed for resource‐limited settings, offering low‐cost, rapid, and sensitive analysis by detecting observable color changes in response to external stimuli. A variety of materials with large surface area and good biomolecule immobilization ability^[^ ^91^ ^]^ have been explored for colorimetric biosensors including nitrocellulose membranes,^[^ ^92^ ^]^ filter papers,^[^ ^93^ ^]^ Whatman paper^[^ ^94^ ^]^ and glass nanofiber papers.^[^ ^95^ ^]^ The introduction of nanomaterials with high surface area and unique optical properties such as metallic NPs,^[^ ^96^ ^]^ CNTs, and graphene oxide (GO)^[^ ^97^ ^]^ have significantly improved the sensitivity and stability of these sensors. Unlike traditional systems that rely on visual detection of color changes, smartphone‐integrated biosensors leverage built‐in cameras and dedicated applications to deliver real‐time results with improved accuracy and reliability. Recently, there has been an increasing interest in empowering smartphone‐based biosensors with image processing and AI algorithms, enabling robust, low‐cost, and rapid analysis that is user‐friendly and accessible to non‐expert users. Sensor systems integrated with AI have the potential to self‐learn from data and make automatic decisions.^[^ ^98^, ^99^ ^]^ Additionally, AI‐integrated colorimetric biosensors can enable personalized health monitoring and be easily used at home by individuals of all age groups. So far, AI has been adopted in various ways to analyze the color change in colorimetric assays including classification, segmentation, and regressions. Recently, Mercan et al. developed an ML‐assisted portable smartphone platform for the robust detection of glucose in saliva using a microfluidic paper‐based analytical device (μPAD). The platform was designed to be adaptable to various lighting conditions and camera optics through image processing and ML classifiers, demonstrating a classification accuracy of 98.24%.

Fluorescence is a phenomenon in which a fluorophore absorbs light at a specific wavelength and emits it at a longer wavelength.^[^ ^100^ ^]^ Fluorometric biosensors, a subclass of optical sensors, operate based on the detection of fluorescence resulting from a biorecognition event.^[^ ^101^ ^]^ Since the 1980s, advances in fluorescence probe technology have driven the widespread adoption of fluorometric biosensors.^[^ ^102^ ^]^ Similarly, the integration of AI into fluorometric biosensors has led to transformative improvements by addressing limitations posed by sample heterogeneity, interference from ambient light, and background fluorescence. Wang et al. integrated transfer learning (TL) into a fluorescent lateral flow assay (LFA) for quantitative detection of upconverted NPs (UCNP) in small datasets without special pre‐processing. TL leverages pre‐trained models and addresses the issue of requiring extensive labeled datasets for AI‐integrated biosensors. Although UCNP‐LFAs are broadly used in medical diagnostics and environmental monitoring, they suffer from low light efficiency and image noise. The study demonstrated that the training time of AI can be significantly reduced with TL and some AI algorithms achieved 100% accuracy with high noise tolerance. The TL‐integrated system reduced hardware requirements which are critical to reducing the cost of analysis and can be extended as a general detection method for optical biosensors.^[^ ^103^ ^]^

SERS is a non‐destructive optical detection technique discovered in 1974. It enables the detection of single molecules by enhancing the Raman signal with electromagnetic and chemical enhancements, making it ideal for biomedical applications. SERS substrates, often engineered with metallic nanostructures, enhance Raman signals to enable ultra‐sensitive biomolecule detection.^[^ ^104^ ^]^ Combined with AI algorithms, these substrates extract spectral features and detect molecular variations associated with diseases such as cancer and infectious diseases, achieving high diagnostic accuracy and specificity. Low et al. used ML with feature selection and dimensionality reduction methods to interpret and analyze high‐dimensional SERS fingerprint spectra for accurate acute myocardial infarction (AMI) diagnosis and prognosis. The ML‐integrated system enabled the measurement of cardiac troponin I, high‐density lipoprotein, and low‐density lipoprotein biomarkers with a high level of accuracy, demonstrating remarkable performance in diagnosing both early and recurrent AMI. In another study by Yang et al. AI was integrated with a SERS biosensor for rapid and low‐cost detection of SARS‐CoV‐2 RNA in human nasopharyngeal swab specimens.^[^ ^105^ ^]^ The biosensor, based on an Ag nanorod array substrate functionalized with DNA probes, used an RNN‐based DL to classify specimens with 98.9% overall accuracy. In blind testing of 72 specimens, 97.2 and 100 % accuracies were achieved for positive and negative specimens, respectively. All detections were completed within 25 minutes, demonstrating that this platform can be used as a rapid and reliable COVID‐19 diagnostic method.

#### 2.2.2 Electrochemical Detection

Detection in electrochemical biosensors relies on biorecognition coupled charge transfer reactions taking place on an electrode‐solution interface. They are broadly categorized into potentiometric, impedimetric, and voltammetric/amperometric biosensors.^[^ ^106^ ^]^ Various studies have shown that AI can enhance their selectivity and overall performance. Zhao et al. developed an innovative AI‐supported voltammetric biosensor for the rapid, low‐cost, and high‐sensitivity detection of insulin and glucose in serum.^[^ ^107^ ^]^ Voltammetric/amperometric biosensors determine the concentration of analytes by measuring electrochemical current from redox reaction following a biorecognition event.^[^ ^108^, ^109^ ^]^ Unlike traditional methods, ML algorithms were used to analyze data obtained from cyclic voltammetry (CV) to accurately separate the signals of two molecules and estimate their concentrations. To train ML algorithms, a large dataset of CV curves was prepared with seven features based on voltage, current, and curve area. The trained models accurately predicted the concentrations of both molecules within minutes. The adaptability and general applicability of the trained model were successfully demonstrated with different serum samples.

Impedimetric biosensors enable label‐free detection of target molecules by analyzing impedance (resistance to current flow) over a range of frequencies using electrochemical impedance spectroscopy (EIS).^[^ ^110^ ^]^ Shahub et al. reinforced an impedimetric biosensor made on a flexible nano‐porous substrate with ML for the detection of cortisol over time via passive sweat collection.^[^ ^111^ ^]^ The sensor successfully measured low levels of cortisol (8–140 ng/mL) in synthetic sweat and ML algorithms classified increasing and decreasing cortisol trends with 100% accuracy during validation tests. Comparable success was achieved with human sweat samples, confirming its validity for real‐life applications.

Ion‐selective electrodes are potentiometric sensors that measure potential differences caused by ions with little or no current flow and stand out with their simplicity, fast response, and resistance to interference.^[^ ^112^ ^]^ Mou et al. combined a magneto‐controlled potentiometric DNA aptasensor with DL to enable classification and quantification of different small molecules in a fast and reliable way.^[^ ^113^ ^]^ Aptamers specific to target antibiotics were immobilized onto magnetic beads to modulate extraction and binding reactions through the application of a magnetic force on an ion‐selective electrode. To demonstrate the classification ability of the proposed system, a potentiometric response dataset (5 aptamers x 13 targets x 3 replicates) was prepared to train and test advanced DL algorithms. According to the results, linear discriminant analysis successfully classified all target molecules with 97.4% accuracy. Furthermore, a special focus was placed on the LSTM neural network to develop a DL algorithm that can extract optimal features to identify and quantify two or more analytes, thus overcoming the limitations of linear models. The algorithm successfully distinguished and quantified five different types of antibiotics with 96.2% accuracy and demonstrated excellent performance in artificial and coastal seawater samples. Classified as another type of potentiometric sensor, field‐effect transistor (FET) biosensors operate based on changes in the surface potential due to interactions with target molecules. In a recent study by Choi et al., Prostate Imaging Reporting and Data System (PI‐RADS) score and the signal of a dual‐gate FET (DGFET) sensor that detects trace amounts of urinary exosomal biomarkers were analyzed by explainable AI.^[^ ^114^ ^]^ The study aimed to assist clinicians in decision‐making and enhance the accuracy in detecting prostate cancer, particularly in ambiguous PI‐RADS 3 lesions. PI‐RADS is a scoring system used to assess the likelihood of prostate cancer in multiparameter magnetic resonance imaging and has a diagnostic accuracy of no more than 30–40%. The results revealed that AI algorithms successfully analyzed the sensor data from multiple biomarkers in conjunction with the PI‐RADS score, increasing the diagnostic accuracy for prostate cancer in PI‐RADS 3 patients from 30.4% to 66.7%.

## 3 Materials for AI‐Enhanced Biosensors

### 3.1 Functional Materials for Biosensing

Functional materials used in biosensing are mainly carbon‐based (e.g., graphene, CNTs), inorganic (e.g., metal oxides, quantum dots [QDs]), and composite materials, each offering distinct advantages.

Carbon‐based materials are widely used due to their high electrical conductivity, large surface area, and ability to enhance fluorescence or electrochemical responses. Inorganic materials, on the other hand, increase the detection sensitivity of biosensors through catalytic and signal amplification. Composite materials combining organic and inorganic components are specifically designed to provide synergistic effects and improve the mechanical strength, flexibility, and stability of portable biosensor platforms. The integration of these materials into AI‐based biosensors has led to significant advances in the fields of disease diagnosis, environmental contaminant detection, and food safety monitoring. By combining innovations in materials science with AI‐based data processing methods, these biosensors enable fast, precise, and decentralized diagnostics, advancing next‐generation smart sensing technologies.

**Table** **1** provides an overview of the materials used in AI‐based biosensors. A variety of materials, including composite, carbon‐based, and inorganic materials, have been used for diagnostic and monitoring purposes. Carbon‐based materials, such as CNTs,^[^ ^121^ ^]^ graphene,^[^ ^130^, ^134^ ^]^ and carbon QDs,^[^ ^138^, ^139^ ^]^ are widely used for their high conductivity, stability, and ability to enhance the performance of fluorescence or electrochemical detection methods. Inorganic materials, such as CuZn‐N bimetallic NPs,^[^ ^119^ ^]^ Au/Ge/SiO_2_,^[^ ^116^ ^]^ and titanium‐based MXenes^[^ ^135^ ^]^ are employed in biosensing platforms because of their catalytic, sensing, and amplification properties. These materials have enabled the detection of biothiols and viruses (spike proteins), along with posture monitoring.^[^ ^116^, ^119^, ^135^ ^]^ They also support a broad range of applications, including colorimetric and fluorescence detection, as well as piezoresistive and electrochemical impedance analysis, offering innovative solutions for real‐time health assessment and disease diagnosis.

**Table 1:** Materials used in AI‐based biosensors.

AI Model	Materials Name	Description	Role in Biosensors	Biosensor Type	Target Analyte	Refs.
CNN/DNN	DeepLactate µPAD	Wax‐printed paper modified with horseradish peroxidase, lactate oxidase, and tetramethylbenzidine	Enables enzymatic colorimetric detection of lactate	Colorimetric	Lactate in sweat	[24]
	PEdELISA microarray	Fluorescence color‐encoded magnetic beads coated with antibodies	Enables multiplexed fluorescence detection	Fluorescence	Serum cytokines	[115]
	Gires‐Tournois (Au/Ge/SiO_2_)	A resonator with porous lossy Ge thin film layer	Increases bioparticle visibility in bright‐field microscopy and maximizes chromatic contrast	Optical	NPs with spike protein	[116]
	QDs	Conjugated with aptamers specific to foodborne pathogens	Provides high fluorescence intensity and stability in a microfluidic device	Fluorescent	*E.coli*	[117]
	PDA NPs	Polydopamine‐based lateral flow immunoassay (LFIA) for quantifying neutralizing antibodies	Serve as LFIA probes due to strong visible light absorption	Colorimetric	COVID‐19	[118]
	CuZn‐N SAzymes	A bimetallic single‐atom nanozyme with high peroxidase‐like activity	Biothiols inhibit CuZn‐N single‐atom nanozyme–catalyzed TMB oxidation and color change	Colorimetric	Biothiol	[119]
	Graphene/SiO_2_	A monolayer graphene coated on SiO_2_ to form a resonator	Graphene served as the active sensing element whereas SiO_2_ provided stability	Optical / SPR	COVID‐19	[120]
	Anti‐BNP conjugated CNT‐TF	Single‐walled carbon nanotube network bridging Au electrodes on a PET substrate	Transduces BNP binding interactions into electrochemical impedance signals	Electrochemical	B‐type natriuretic peptide (BNP)	[121]
Tree‐based ensemble methods	3DP‐IDE‐CBPE‐ECL	3D‐printed structure with aptamer, enzyme, and antigen as bioreceptors	Enables electrochemiluminescence (ECL)‐based detection of multiple biomarkers	ECL	Glucose Lactate Choline	[122]
	3D Printed SE‐ECL	3D‐Printed Single‐electrode ECL platform	Redox species generation and optical emission induction via Luminol/H_2_O_2_ electrochemistry	ECL	Glucose Lactate	[123]
	Vmh2‐H3w	Hydrophobin‐based chimera (Vmh2‐H3w) immobilized on polystyrene multiwell plates, functionalized with GFP as a fluorescent probe	Molecular recognition, stable immobilization, efficient fluorescent signal transduction	Fluorescence	Mercury	[124]
	ZnO nanoflowers	Synthesized by a mild hydrothermal method	High isoelectric point and surface coverage contributed to high sensitivity	Electrochemical	Cardiac troponin‐I and T	[125]
	f‐AuNPs	Hydrophobin chimera (Vmh2‐GKY20) functionalized AuNPs	f‐AuNPs form aggregates with bacteria, causing change in color intensity	Colorimetric	*E. coli S. epidermidis*	[126]
Statistical and traditional ML techniques	LC‐based aptasensor	Liquid crystalline mixture (E7)	E7 liquid crystal increased sensitivity to surface interactions biomolecular interactions revealed in optical microscope	Optical	*E. coli*	[127]
	2D PC biosensor	2D photonic crystal structure on a silicon‐on‐insulator substrate, triangular lattice with holes	Photonic Crystal‐Based Sensing	Optical	Cancer cells (HeLa, PC12, MDA, MCF, and Jurkat)	[128]
	Microneedle patch	Silk fibroin methacryloyl hydrogel with PVA, MOF (Bi–PCN–222), and curcumin	Provides antimicrobial properties, real‐time wound pH monitoring	Fluorescence	Bacterial infections, Wound pH	[129]
	GF/Ni/Anti‐LCN2 + ELM	A 3D graphene electrode functionalized with lipocalin‐2 antibody used for neutrophil gelatinase‐associated lipocalin (NGAL) detection	Detects NGAL levels for early acute kidney injury prediction	Electrochemical	NGAL in urine	[130]
	µPAD	A paper‐based sensor platform with zones (I‐IV) for reaction. Wax valve to control fluid flow.	Serves as a colorimetric detection platform, where the reaction between potassium ferricyanide and ferric chloride in Zone III generates Prussian blue	Colorimetric	Uric acid in saliva	[131]
	CIP	Electrochemically synthesized cell‐imprinted polymer using the monomer 3‐aminophenylboronic acid with a phenylboronic acid group	Forms more selective template binding sites with phenylboronic acid for identification and capture of target bacteria	Electrochemical	*E. coli S. aureus V. parahaemolyticus*	[132]
Statistical and traditional ML techniques	RamR	Evolved RamR used as a protein‐based biosensor in microbial fermentation; sensitive to 4′‐O‐methylnorbelladine (4NB)	Discriminates between 4NB and its precursor norbelladine; used to monitor Nb4OMT enzyme activity	Fluorescence	4NB (microbial metabolite)	[133]
	GSSRR and GSRRTW	Graphene‐based single split ring resonator (GSSRR) and graphene‐based single split ring resonator with thin wire (GSRRTW)	To optimize the light absorption characteristics of the biosensor and detect small changes in the refractive index with high sensitivity, the graphene‐supported metasurface structure was used as part of GSSRR.	Refractive Index	Hemoglobin	[134]
	N‐(MX/GNR) hybrids	2DTi_3_C−2Tx MXene/1D nitrogen‐doped graphene nanoribbon hybrid	Piezoresistive pressure sensing layer	Pressure sensor	Pressure variations	[135]
	μPAD *c*‐ELISA	Smartphone‐controlled μPAD with pre‐stored reagents and paperrotary valves	Enables automated detection	Colorimetric	β‐amyloid peptide 1–42	[136]
	Peptide‐conjugated PS	Polystyrene particles coated with bacteria identifying short peptides	Interaction of peptides on PS with bacteria alters aggregation, surface tension, viscosity, and flow velocity through paper pores.	Physical (flow velocity)	*E.coli S. aureus S. Typhimurium E. faecium P. aeruginosa*	[137]

**Figure 3:**
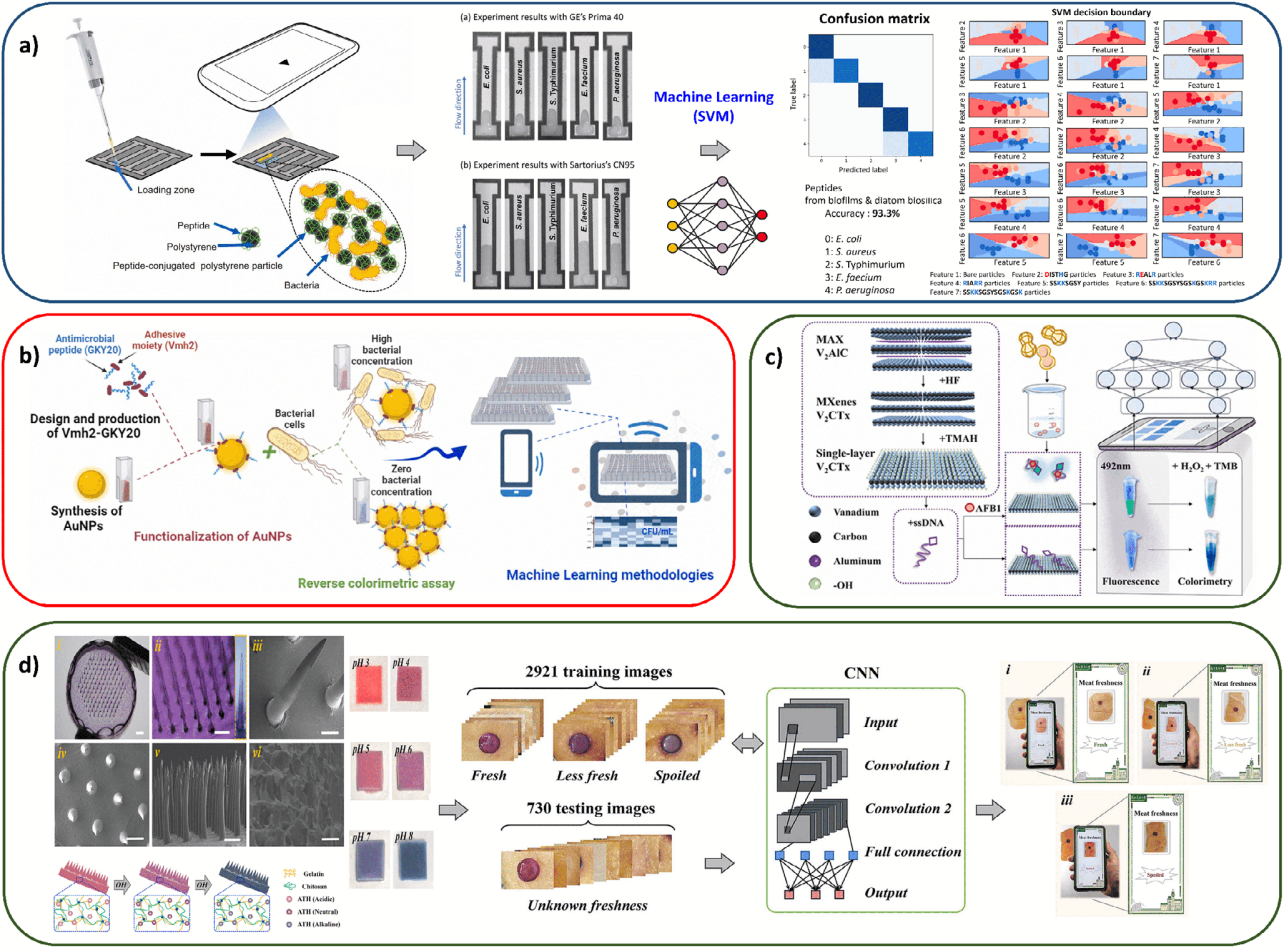
Recent innovations in biosensing materials across various application areas. A paper microfluidic chip for AI‐assisted classification of five different bacterial species based on the flow velocity of mixtures containing target bacteria and biofilm‐derived short peptide conjugated polystyrene particles (a). Reproduced with permission,^[^ ^137^ ^]^ Copyright 2021, Elsevier. Hydrophobin‐chimera functionalized AuNPs for AI‐assisted colorimetric assessment of bacterial contamination (b). Reproduced with permission^[^ ^126^ ^]^ Copyright 2024, Elsevier. An AI‐assisted dual mode colorimetric/fluorescence aptasensor constructed using V_2_C Mxene nano‐enzyme to detect AFB_1_ (c). Reproduced with permission^[^ ^151^ ^]^ Copyright 2024, Elsevier. A colorimetric microneedle sensor comprising pH‐responsive anthocyanins for AI‐assisted meat freshness assessment (d). Reproduced with permission^[^ ^153^ ^]^ Copyright 2024, Elsevier.

Materials used in environmental applications can be classified as inorganic and/or composite. Inorganic materials including metal oxides like RuO_2_, La_0.87_Sr_0.13_CrO_3_, and In_2_O_3_ are preferred for catalytic applications in addition to sensing, unlike semiconductors such as indium tin oxide (ITO).^[^ ^140^, ^141^ ^]^ Composite materials combine inorganic and organic components to enhance sensor performance, such as AuNPs functionalized with hydrophobin chimeras or aptamers for pathogen detection, peptide conjugated polystyrene particles for bacterial species classification, or the combination of GO and ionic liquids to improve conductivity, sensitivity, and stability for pollutant detection (**Figure** **3**a,b).^[^ ^126^, ^137^, ^142^ ^]^ Similarly, various materials have been employed in AI‐based biosensors for food safety and agriculture applications to detect spoilage, contaminants, and pathogens in food. These biosensors often incorporate composite materials like chitosan NPs embedded in cellulose acetate (CA), which are used for colorimetric sensing, as seen in the detection of amine gases from meat spoilage.^[^ ^143^ ^]^ MOFs combined with substrates such as chitosan and polyvinyl alcohol enable real‐time detection of spoilage gases.^[^ ^144^, ^145^ ^]^ Additionally, materials like functionalized AgNPs,^[^ ^146^ ^]^ AuNPs,^[^ ^147^ ^]^ magnetic NPs,^[^ ^148^, ^149^ ^]^ and CNTs^[^ ^150^ ^]^ have been reported to enhance sensitivity and accuracy in AI‐assisted detection of H_2_O_2_ for freshness, antibiotics, bacteria, and toxins in food. Materials like 2D MXenes and black phosphorene, along with fluorophores, have been integrated into AI‐assisted fluorescence and electrochemical sensors for detecting contaminants such as aflatoxin and herbicide (Figure 3c).^[^ ^151^, ^152^ ^]^ Furthermore, anthocyanin‐loaded hydrogels, hydroxypropyl methylcellulose, and fluorescein‐based films have also been used for fluorescence and colorimetric monitoring of volatile nitrogen‐based gases from spoiled seafood and meat.^[^ ^153^, ^154^, ^155^ ^]^

### 3.2 Emerging Material Innovations

Recent advancements in AI‐based biosensor materials have led to the development of innovative sensing platforms with enhanced sensitivity, flexibility, and multi‐functionality. Notable examples include paper‐based sensors, 3D‐printed materials, liquid crystal (LC)‐based aptasensors, and microneedle patches. For instance, an AI‐optimized 3D‐printed electrochemiluminescence biosensor (3DP‐IDE‐CBPE‐ECL) has integrated aptamers, enzymes, and antigen receptors to facilitate multi‐biomarker detection, including glucose, lactate, and choline.^[^ ^122^ ^]^ Similarly, an AI‐assisted microfluidic biosensor utilizing lectin concavalin A and aptamer‐conjugated QDs for enrichment and sensitive fluorescence detection, respectively, detected E. Coli at very low concentrations and showed remarkable performance in milk and chicken samples, highlighting its potential for broad application in foodborne pathogen detection and food safety analysis.^[^ ^117^ ^]^ A DL‐integrated μPAD, which uses wax‐printed paper modified with horseradish peroxidase and lactate oxidase for enzymatic colorimetric lactate detection in sweat, offers a cost‐effective and disposable alternative.^[^ ^24^ ^]^ Furthermore, an ML‐assisted LC‐based aptasensor has been reported as a promising approach for microbial detection. The sensor provided molecular recognition properties of LC mixtures to detect *E. coli* with high specificity.^[^ ^127^ ^]^ Additionally, combined with a DL‐integrated colorimetric microneedle sensor (CMS), edible anthocyanin‐loaded hydrogel (gelatin, chitosan, carboxymethyl cellulose) has provided a non‐invasive approach for monitoring pH variations in meat tissue fluids, aiding in food freshness assessment (Figure 3d).^[^ ^153^ ^]^ These emerging AI‐based biosensing technologies highlight the growing integration of advanced materials in biosensors, driving developments in real‐time, portable, and highly sensitive detection in healthcare, environmental monitoring, and food safety applications.

## 4 Sector‐Specific Applications of AI‐Assisted Biosensors

**Figure 4:**
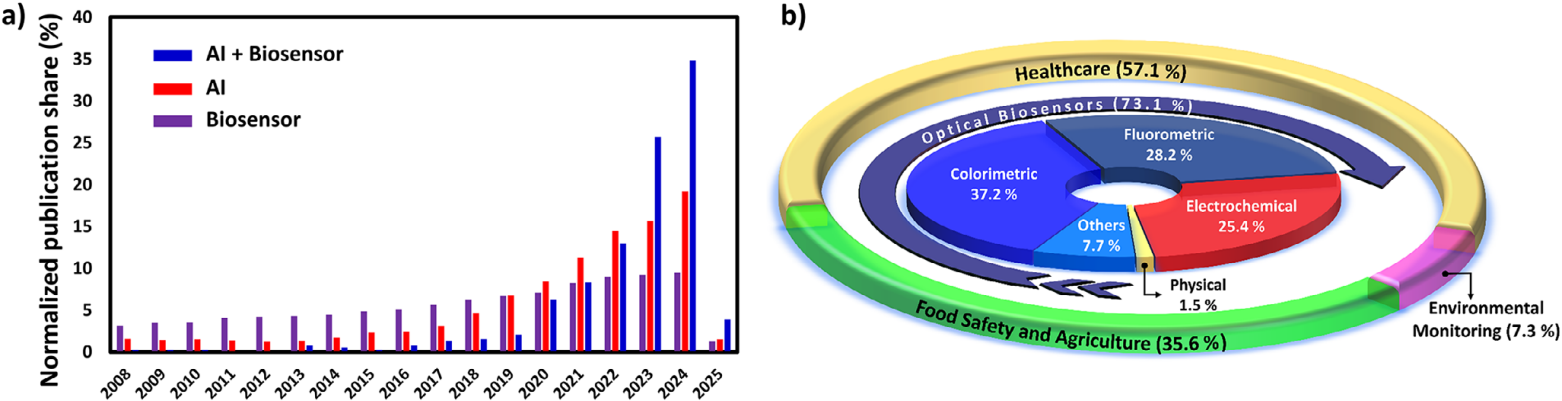
The percentage of papers on AI, biosensors, and AI‐assisted biosensors published between 2008 and 2025 (a). The distribution of papers on AI‐assisted biosensors categorized by detection method and application (b).

A Web of Science search was conducted for the papers published since 2008 with the keywords “artificial intelligence,” “biosensor,” and “artificial intelligence + biosensor.” The papers found with the keyword combination “artificial intelligence + biosensor” contained both keywords together. The percentage distribution over the years was calculated for each keyword group (i.e., the number of articles containing the keyword(s) in a given specific year divided by the total number of articles containing the same keyword(s) since 2008). The normalized publication share results indicated a rising trend in AI‐related papers, with the steepest increase observed in AI‐assisted biosensors, reflecting a growing interest in this field (**Figure** **4**a). A statistical analysis of publications on AI‐assisted biosensors since 2020 has revealed that optical detection methods were the most used with 73.1%, followed by electrochemical (25.4%) and physical detection (1.5%), which can be attributed to the high‐level compatibility of AI with image‐based data (Figure 4b). Furthermore, most papers focused on healthcare (57.1%) and food safety & agriculture (35.6%), highlighting the research gap in environmental monitoring. **Table** **2** lists recent papers on applications of AI‐assisted biosensors across various sectors.

**Table 2:** AI algorithms utilized in biosensors across different sectors and their performance metrics.

	Target Analyte	Detection Method	AI Algorithm	Accuracy/Error	LOD	Linear Range	Refs.
Healthcare	Hemoglobin	Optical	ML‐based PR	Sensitivity 2000 nm/RIU Accuracy ≈ 1.0 R^2^	–	–	[134]
	Glucose (Glu) Lactate (Lac)	Electrochemical	LR, DT, k‐NN AdaBoost	R^2^ (Glu): 98% R^2^ (Lac): 99%	Glu: 0.04 mM Lac: 0.1 mM	Glu: 0.1 ‐ 1 mM Lac: 0.1 – 4 mM	[123]
	Buprenorphine	Electrochemical	ML‐based LR	R^2^: 0.9832	0.129 μM	2 to 140 μM	[179]
	B‐type natriuretic peptide	Electrochemical	DNN (Classification)	–	–	–	[121]
	COVID‐19	Optical	DL‐based ResNet 50	High IoU	160 ng mL^−1^	625–10000 ng mL^−1^	[118]
	Glu Lac Choline (Cho)	Electrochemical	DL ‐ YOLOv3 ML‐based RLR, SVM, DT, RF, GB, k‐NN	R^2^ (Glu) 95%/MSE: 0.17 R^2^ (Lac): 90%/MSE: 0.55 Cho ‐ R^2^: 91% /MSE: 0.1	Glu: 0.033 mM Lac: 0.07 mM Cho: 0.0007 mM	Glu 0.05 ‐ 3 mM Lac 0.1–4 mM Cho 0.0007–1 mM	[122]
	Uric Acid	Optical	ML‐based LR, MR, PR, MPR, SVR, DTR, RFR	MAE: 4.242 ppm	–	–	[131]
	Lac	Optical	DL‐based MobileNet, Xception, VGG19, VGG16, ResNet50, Inception‐v3	Accuracy (Inception‐v3): 99.9%	0.67 mM	–	[24]
	Blood alcohol	Physical	ML‐based ET	–	–	–	[166]
	Aβ 1–42	Optical	DL‐based YOLOv5	Accuracy: 97% Specificity: 98%	10.07 pg mL^−1^	–	[136]
	Glu	Optical	Ensemble bagging classifier MLR	R^2^:0.95 R^2^: 0.97	25 μM	0.25 μM‐ 3 mM 3–30 mM	[170]
Environmental Monitoring	Water Methanol Ethanol	Electrochemical	ML‐based LR	–	–	–	[140]
	Methane	Electrochemical	ANN	Accuracy: 98%	1000 ppm	–	[141]
	Hg^2 +^	Optical	ML‐based MLP RF, XGB	Accuracy: 95% for UVc 85% for PUVc	Tap water: 0.4 nM Sea water: 0.3 nM	–	[124]
	DEHP BPA	Electrochemical	ML	–	DEHP: 0.58 pg mL^−1^ BPA: 0.59 pg mL^−1^	–	[142]
	Pb^2 +^	Optical	ML‐LR, NLR	LR ‐ RMSE: 0.1754 NLR ‐ RMSE: 0.1244	0.5 ppb	0.5‐2000 ppb	[180]
Food safety and Agriculture	*S. Typhimurium*	Optical	Region Based‐CNN	Accuracy: 73% and 97.24%	55 CFU mL^−1^.	6.9 × 10^1^ to 1.1 × 10^3^ CFU mL^−1^	[181]
	MH	Electrochemical	ML‐based LS‐SVM, ANN	–	0.3 μM SPE 0.1 μM GCE	0.7–55 μM SPE 0.3–600 μM GCE	[152]
	NH_3_ Methylamine Trimethylamine Putrescine Cadaverine	Optical	CNN	98.5%	–	–	[143]
	Target Analyte	Detection Method	AI Algorithm	Accuracy/Error	LOD	Linear Range	Refs.
	Amine	Optical	CNN	Accuracy (WISeR50): 98.95%	80 ppm	–	[145]
	Anthocyanins	Optical	ML‐based RF	Accuracy: 98.8%	7.31 mg N/100 g	–	[155]
	Amine gases	Optical	CNN	Accuracy(WISeR50): 99.94%	Ammonia 37.17 ppm Methylamine 25.90 ppm Trimethylamine 40.65 ppm		[144]
	Nitrogen	Optical	Back Propagation‐NN	–	–	–	[182]
	Anthocyanins	Optical	DL‐based VGG19	95.3 %	–	–	[153]
	Volatile organic compounds	Optical	ML‐based sPLS‐DA svmLinear svmRadia RF, ANN, HDDA	0.83	–	–	[183]
	AFB1	Optical	ANN	R^2^: 0.99	Fluorescence: 0.0905 ng mL^−1^ Colorimetric: 0.6845 ng mL^−1^	Fluorescence: 0.1–500 ng mL^−1^ Colorimetric: 1–800 ng mL^−1^	[151]
	*E. coli*	Optical	ANN XGB	Water sample (R^2^: 0.986 and RMSE: 0.209) Juice sample (R^2^: 0.976 and RMSE: 0.262)	6 CFU mL^−1^	–	[127]
	*E. coli*	Optical	Classification & Regression ML‐based RF, XGB, MLP, SVM, PLS	Accuracy: 97% RMSE: 0.04	10 CFU mL^−1^	–	[126]
	*S. Typhimurium*	Optical	ML‐based LR	R^2^: 0.97	40.5 CFU mL^−1^	50‐10^7^ CFU mL^−1^	[149]
	*C. jejuni E. coli L. monocytogenes S. Typhimurium*	Optical	ML‐based k‐NN, DT, SVM, XGB	XGB ‐ Accuracy: 83.75%	–	–	[183]
	Nitrate	Electrochemical	SVM	R^2^: 97% MSE: 0.036	–	–	[184]
	Estradiol	Optical	ML‐based LR, RFR SVR, ANN	Training set ‐ R^2^: 0.963 Test set ‐ R^2^: 0.976 Validation set ‐ R^2^: 0.991 Entire dataset ‐ R^2^: 0.970	9.3 × 10^−7^ µg mL^−1^	1 × 10^−6^–1 µg mL^−1^	[185]
	*E. coli*	Optical	RF, MDF	Accuracy Compound identification: 65% Class classification: 90%	–	125 ppb to 1000 ppb	[186]
	CAP KAN NEO	Optical	Partial least squares regression	Accuracy: >99 %	CAP 0.03 ng mL^−1^ KAN 0.07 ng mL^−1^ NEO 0.4 ng mL^−1^	pg mL^−1^ to ng mL^−1^m	[148]
	KAN AMP OTC SMX	Optical	SVM	–	KAN: 37.5 ppb AMP: 1 ppb OTC:25 ppb SMX: 6.25 ppb	–	[147]
	Dichlorvos	Optical	ML	97.6%	0.65 μM	1–7 μM	[187]
	Nitrate	Electrochemical	ANN	R^2^: 0.90 MSE: 0.0448	–	–	[188]

### 4.1 Healthcare

Recently, the integration of AI and biosensor technologies has made significant progress in the field of healthcare and the monitoring of both invasive and non‐invasive biological parameters. This section provides an analysis of recent advancements in AI‐assisted non‐invasive and invasive biosensors.

#### 4.1.1 Non‐Invasive Biosensors

Non‐invasive biosensors are devices that operate outside the body without any invasive procedure and collect biological data in bodily fluids such as saliva, sweat, or tears.^[^ ^156^, ^157^, ^158^, ^159^ ^]^ Their painless and low‐risk structure could contribute to health awareness and make healthcare services more accessible. Advances in biomaterials, microelectronics, and nanotechnology have significantly improved the reliability, sensitivity, and portability of non‐invasive biosensors, enabling their integration into wearable devices such as fitness bands, smartwatches, and skin‐patch sensors. The rising demand for personalized healthcare along with rapid advancements in the field attracts the attention of companies seeking to commercialize such biosensors.^[^ ^160^ ^]^ A notable example would be GlucoWatch, an integrated wristwatch approved by the United States Food and Drug Administration, which employs reverse iontophoresis to extract ISF from the skin for glucose measurement.^[^ ^161^ ^]^ The high demand for remote healthcare services during the COVID‐19 pandemic has revealed the importance of these sensors in chronic disease management and early diagnosis. Non‐invasive biosensors generally benefit from cloud‐based data management systems and wireless communication technologies such as Bluetooth and Wi‐Fi for real‐time data collection, and analysis, which could enable personalized healthcare plans.^[^ ^162^, ^163^ ^]^ The integration of smartphones as readers has further accelerated their adoption by making these innovations more accessible and simplifying data analysis and visualization.^[^ ^164^, ^165^ ^]^ AI‐assisted smartphone applications can analyze large volumes of sensor data and provide instant feedback. For instance, Fairbairn et al. applied ML to a commercial smartphone‐integrated transdermal sensor for real‐time quantification of blood alcohol concentration by analyzing high‐frequency sensor data.^[^ ^166^ ^]^ Time series feature extraction and ML significantly increased prediction accuracy compared to sensors without AI assistance. The use of ML‐enabled an effective assessment of drinking episodes, highlighting its potential for real‐world applications and addiction science.

 μPADs are frequently used in the development of non‐invasive biosensors as they meet the criteria set by the World Health Organization for new point‐of‐care diagnostic tests including affordability, sensitivity, specificity, user‐friendliness, rapidity, robustness, equipment‐free operation, and easy delivery.^[^ ^167^, ^168^, ^169^ ^]^ Additionally, features such as low‐cost, environmental friendliness, simple structure, and no need for external equipment make μPADs an ideal device for delivering health services to large populations. AI has been used to identify complex patterns and correlations in raw data of μPADs that are difficult to detect manually, thereby enhancing the accuracy, speed, robustness, adaptability, and efficiency of analysis. Ghateii et al. integrated a µPAD with ML to detect glucose in real human plasma serum with a flash/no‐flash technique.^[^ ^170^ ^]^ The proposed system successfully classified and quantified glucose levels across a broad range (1–30 mM) with up to 95% accuracy, highlighting the value of AI in uncontrolled environments. In another study, Yüzer et al. developed a DL‐assisted, offline smartphone system to detect lactate levels in sweat accurately. To train various DL algorithms, a large dataset (10 080 images) was created by capturing color changes induced by enzymatic conversion of lactate on μPAD patches using different smartphone cameras and lighting conditions. Following an image processing step, the dataset was used to train and test six different CNN models. To provide a user‐friendly interface and offline analysis, the best‐performing model (Inception‐v3, with 99.9% accuracy) was embedded into a smartphone application. The integrated system demonstrates a broad application potential in various fields, from sports medicine to personal health monitoring.^[^ ^24^ ^]^ In a similar approach, Liu et al. integrated ML with μPADs to colorimetrically quantify uric acid (UA) levels in saliva.^[^ ^131^ ^]^ The algorithm that predicts lactate in extracted features consists of relevant region detection, color calibration, feature extraction, and feature analysis with ML. In artificial saliva samples, the DT model gave the best results with an average error of 4.2 ppm, and a high correlation (r = 0.6140, p<0.0001) was found between the estimated salivary and blood UA in clinical samples. This method offers an accurate and sensitive result compared to commercial test strips. Furthermore, Basturk et al. integrated a DL‐based regression model with a μPAD for colorimetric quantification of glucose, cholesterol, and pH levels in tears.^[^ ^171^ ^]^ In the study, several AI regression models were trained with images taken under seven different lighting conditions with smartphones of four different brands, and the best‐performing model was integrated into a smartphone application, enabling rapid, offline, and accurate quantitative measurement of analytes. The system was able to analyze three analytes in physiological concentration ranges within minutes with a low RMSE. AI‐assisted biosensors with a smartphone application can also provide real‐time health monitoring and early diagnosis for personalized health monitoring applications. In a study by Sittihakote et al., a 3D graphene‐based electrochemical immunosensor was combined with an Extreme Learning Machine (ELM) algorithm for accurate diagnosis and near‐real‐time monitoring of acute kidney injury (AKI).^[^ ^130^ ^]^ The biosensor was designed to electrochemically detect Neutrophil Gelatinase Associated Lipocalin (NGAL), a biomarker that plays an important role in the early diagnosis of AKI, in human urine. The use of AI increased the accuracy of the biosensor from 82.96% to 91.85% by reducing noise in electrochemical measurements, reduced the margin of error from ±6 ng mL^−1^ to ±0.54 ng mL^−1^, and lowered the LOD from 14.8 ng mL^−1^ to 0.89 ng mL^−1^. In another study, Kumar et al. reported an ML‐assisted 3D printed ECL biosensor to provide a portable, cost‐effective, and real‐time solution for the detection of human metabolites such as glucose and lactate.^[^ ^123^ ^]^ Analyte concentration and ECL signal emission datasets were used to train and test various ML algorithms (LR, DT, RF, k‐NN, and AdaBoost) to predict the levels of target analytes. An Android application was developed to perform operations such as automated real‐time image acquisition, segmentation, data processing, cloud sharing, and ML‐assisted biomarker prediction. The integrated system exhibited excellent accuracy (AdaBoost, 98% accuracy) and low LOD for lactate (0.04 mM) and glucose (0.1 mM), offering a reliable and effective method for monitoring multiple biomarkers in clinical applications.

#### 4.1.2 Invasive Biosensors

Invasive biosensors are typically designed to be implanted or inserted, partially or fully, into the body and operate inside tissues, blood, or interstitial fluids (ISF) to monitor critical biochemical parameters. Although electrochemical detection is the most widely used method due to its simplicity and broad applicability, invasive biosensors may operate based on other detection principles such as optical, magnetic, and piezoelectric.^[^ ^172^, ^173^, ^174^ ^]^ By detecting specific biomarkers, invasive biosensors can be used to improve the diagnosis and treatment of various conditions such as diabetes, sepsis, heart failure, myocardial infarction, and various types of cancer, including prostate and colorectal cancer.^[^ ^175^ ^]^

Given the high prevalence of diabetes, a special focus has been placed on the commercialization of glucose biosensors that monitor blood sugar levels and provide instant feedback to users.^[^ ^176^ ^]^ Beyond individual health management, biosensor technology also has the potential to improve epidemiological surveillance and epidemic control.^[^ ^177^, ^178^ ^]^ The COVID‐19 pandemic, one of the most important challenges faced by global health systems, has revealed the urgent need for innovative and effective technologies to evaluate the efficacy of treatment, manage outbreaks, and ensure the sustainability of health services. Tong et al. developed a platform that integrates LFA technology with AI to quantify neutralizing antibodies and therefore evaluate the effectiveness of COVID‐19 vaccines.^[^ ^118^ ^]^ The platform uses LFA based on polydopamine (PDA) NPs and is coupled with a smartphone‐based reader with a trained network to detect the location of the test lines for potential bias elimination and calculate the target antibody concentrations by analyzing the intensity values.

The results revealed that the integrated system showed high accuracy, consistency and fast response compared to enzyme‐linked immunosorbent assay (ELISA) kits on 30 serum samples. The platform was able to monitor vaccine effectiveness at the individual level, thus supporting efforts to ensure herd immunity. Early diagnosis and effective management of chronic diseases with high mortality and morbidity, such as heart failure, remains a significant challenge in modern healthcare and requires the development of portable and low‐cost diagnostic tools that not only transform individual patient care but also support the healthcare system. Gou et al. applied DNN to EIS data of CNT thin‐film (CNT‐TF) B‐type natriuretic peptide (BNP) biosensors for highly accurate heart failure classification without advanced electronics and computing systems. The proposed approach aimed to address the issue of reproducibility and accuracy in CNT‐based biosensors, which arises from the intrinsic variation of CNTs and limits their real‐world clinical applications. The DNN‐based approach achieved 80% out‐of‐sample accuracy for both PBS and clinical blood samples, surpassing traditional methods. In general, the study shows how AI can effectively address the challenges of sensor data analysis, particularly in situations with noisy data and complex data structures.^[^ ^121^ ^]^

**Figure 5:**
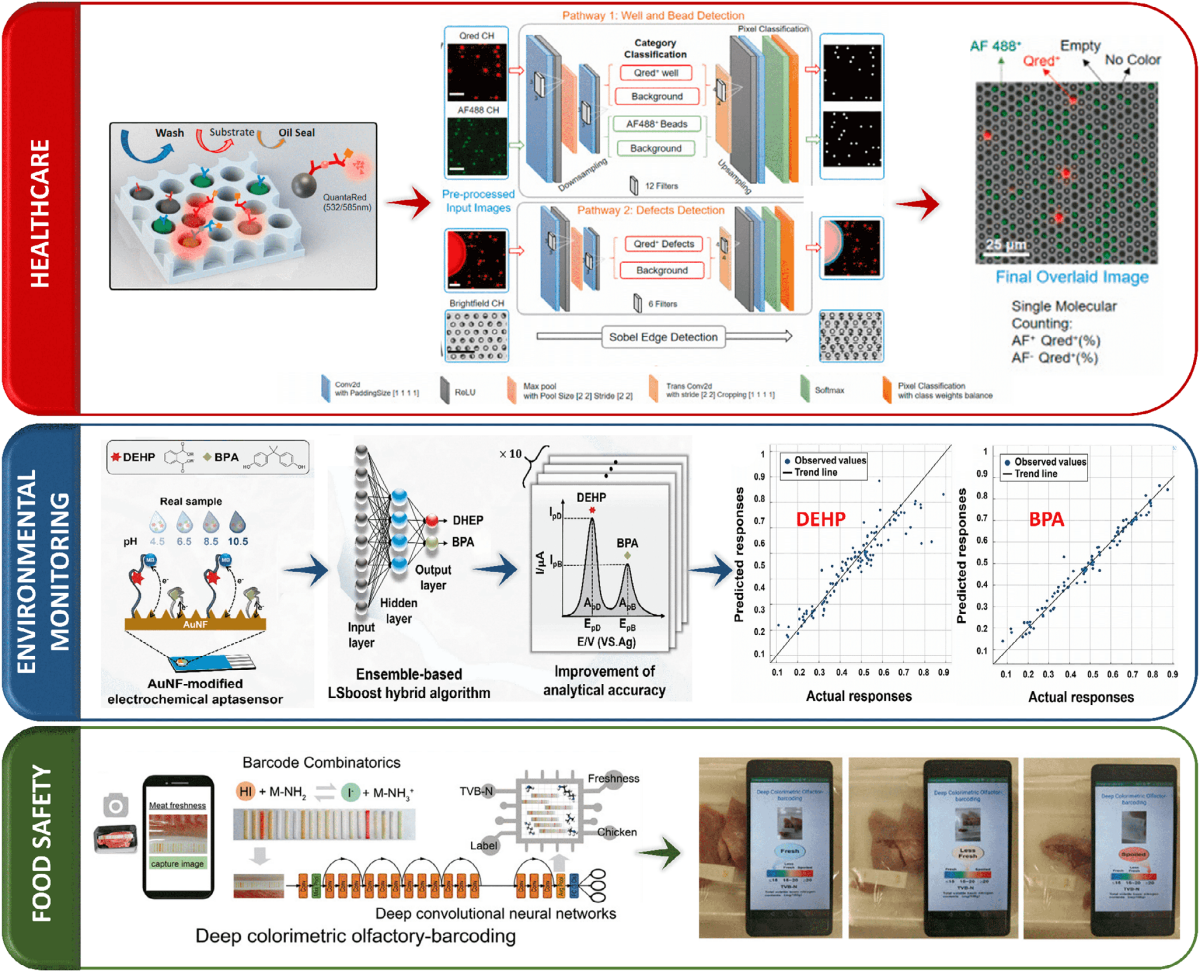
In healthcare, a microarray digital immunoassay platform was combined with a CNN‐based ML algorithm to achieve spatial‐spectral coding and high throughput image processing on a microfluidic chip containing fluorescent color‐encoded magnetic beads to detect cytokines. Reproduced with permission,^[^ ^115^ ^]^ Copyright 2021, Elsevier. In environmental monitoring, ML was integrated with an electrochemical Au nanoflower modified aptasensor to detect trace amounts of DEHP/BPA in 12 rivers with different pH levels. Reproduced with permission,^[^ ^142^ ^]^ Copyright 2024, Elsevier. In food safety, a cross‐reactive colorimetric barcode combinatorics was integrated with DCNNs to create scent fingerprints from porous nanocomposite barcodes, predicting food freshness and enabling real‐time and non‐destructive food freshness monitoring via a smartphone app. Reproduced with permission,^[^ ^143^ ^]^ Copyright 2020, Wiley Online Library.

Detection of multiple analytes in bodily fluids is crucial as it offers a more holistic, accurate, and cost‐effective approach to monitoring health, personalizing treatments, conducting research, and diagnosing diseases. In a study by Song et al., a microarray digital immunoassay platform was combined with a CNN to achieve spatial‐spectral coding and ML‐based image processing on a microfluidic chip (**Figure** **5**).^[^ ^115^ ^]^ The study was conducted to expand the multiplex capacity of the digital immunoassay and achieve high sensitivity in small sample volumes with a short turnaround time of the assay. CNN automatically classified and segmented multicolor fluorescent image features such as microwells, beads, and defects with high throughput, yielding 8–10 times higher accuracy than the standard method based on global thresholding and segmentation. Applied to cytokine profiling in patients with cytokine release syndrome, the platform measured 12 different cytokines from pg/mL to ng/mL in a clinically relevant dynamic range using only 15 µL of serum sample, completing the entire process from sample loading to results in 40 minutes. In addition, CNN performed well in reducing optical crosstalk that would lead to incorrect signal identification in conventional assays. Similarly, Srivastava et al. applied multiple ML regression models including linear regression (LR), segment‐based, and ensemble methods to a 3D‐printed interdigitated bipolar ECL‐based biosensor to achieve reproducible and accurate results in detecting biomarkers such as glucose, lactate, and choline in real blood serum. Although ordinary least squares‐based regression models were easiest to implement, ensemble methods such as DT, RF, and Gradient Boost (GB) produced the best R^2^ values and the lowest error scores. The integrated platform showed promising results when tested with real blood serum.^[^ ^122^ ^]^

In recent years, there has been a surge in published papers on integrating AI algorithms with paper‐based colorimetric sensors for analyzing images, processing data, and interpreting results reliably. Duan et al. applied DL to μPADs for accurate and offline detection of the β‐amyloid peptide 1–42 (Aβ 1–42 peptide), a crucial biomarker of Alzheimer's disease. A color change‐based ELISA (c‐ELISA) was used to detect Aβ 1–42 peptide in artificial plasma samples. Eight smartphones were used to increase the robustness and adaptability of DL algorithms. YOLOv5 DL model, which achieved higher accuracy than traditional curve‐fitting, was embedded into a user‐friendly smartphone application using Tencent's Neural Network Inference Framework for offline DL‐assisted Alzheimer's disease screening.^[^ ^136^ ^]^ Similarly, the use of AI in biosensor technology could offer significant benefits in drug management applications, particularly in mitigating the risks of opioid overdose and addiction. Kadian et al. developed a 3D‐printed conducting microneedle‐based electrochemical sensor and integrated it with ML for economic, wireless, efficient, and selective determination of buprenorphine at micromolar levels in ISF.^[^ ^179^ ^]^ The sensing data was used to train and test a univariate LR model, achieving a good prediction performance with an R^2^ value of 0.985. The trained model was embedded into a web application, allowing end users to easily access and view the prediction results.

In addition to improving the performance of biosensors, AI can also be used to optimize biosensor design and to determine the best combinations of geometric and material parameters. For instance, Patel et al. used AI to design a graphene‐based metasurface refractive index biosensor for highly sensitive detection of hemoglobin.^[^ ^134^ ^]^ Polynomial regression models trained on six parameters, including the angle of incidence, substrate length, substrate width, substrate thickness, graphene chemical potential, and resonator thickness, predicted absorption values for intermediate wavelengths with high accuracy (R^2^ close to 1.0 for polynomial degrees above 5). The study also shows that ML algorithms trained on partially simulated data can successfully predict the remaining design simulation parameters and values.

### 4.2 Environmental Monitoring

Conventional water quality sensors are often limited by factors such as sensitivity, response time, and the complexity of environmental matrices. Integration of AI with biosensors allows for the accurate and cost‐effective detection of waterborne pollutants, even in complex water systems. This integration enables the adaptability of biosensors to varying conditions, such as pH fluctuations, turbidity, or interfering substances, enhancing their reliability. Furthermore, AI‐powered biosensors offer higher sensitivity and faster response times in the detection of contaminants such as heavy metals, endocrine‐disrupting chemicals from the plastic industry, and bacterial pathogens in water bodies.

A graphene field‐effect transistor (GFET) combined with ML, using a Multilayer Perceptron (MLP) classifier, has been utilized to enhance the selectivity of gas sensors.^[^ ^140^ ^]^ By decoupling the conductivity of sensor profiles into distinct physical properties, which are then classified using MLP algorithms, GFET‐based sensors can precisely detect gases like water vapor, methanol, and ethanol, even in complex mixtures. This approach enables miniaturization, low power consumption, and improved selectivity without the need for multiple sensing materials. Another important development in environmental monitoring is a four‐electrode electrochemical sensor designed for methane detection.^[^ ^141^ ^]^ The sensor, composed of La_0.87_Sr_0.13_CrO_3_, In_2_O_3_, Au, and Pt, utilizes yttria‐stabilized zirconia as a solid electrolyte. ANNs were employed to analyze sensor data, enabling the discrimination of methane sources such as cattle, wetlands, and natural gas with over 98% accuracy. High accuracy and portability make the sensor a promising tool for methane emissions monitoring, with applications in environmental conservation and climate change mitigation.

Expanding the scope of ML‐powered biosensing, a fluorescence‐based approach has also been explored to detect heavy metal contaminants such as mercury (Hg^2 +^) in water using a hydrophobin chimera for high specificity and sensitivity, with LODs of 0.4 nM in tap water and 0.3 nM in seawater.^[^ ^124^ ^]^ The integration of ML algorithms such as MLP, RF, and XGBoost with fluorescence data enables accurate prediction of Hg^2 +^concentrations, even in the presence of interfering metals, providing an efficient and affordable in situ monitoring solution for Hg^2 +^ pollution that can be operated by non‐expert personnel through smartphone‐based systems. The same group of authors extended their study to bacterial detection using a chimeric protein functionalized AuNPs (f‐AuNPs) for selective binding.^[^ ^126^ ^]^ A reverse colorimetric assay integrated with ML classifiers, including RF, MLP, XGBoost, SVM, PLS, and their combinations allowed *E. coli* detection at 10 CFU mL^−1^ within 15 minutes, demonstrating its applicability for monitoring bacterial contamination in real‐world samples. Similar AI‐based smartphone applications were explored for peroxide detection^[^ ^189^ ^]^ and quantifying key water quality parameters, including various anions and cations such as ammonium, arsenic, carbonate, chloride, iron, nitrate, and sulfate.^[^ ^29^ ^]^ Solmaz et al. developed a smartphone application for peroxide quantification using colorimetric test strips.^[^ ^189^ ^]^ A Cloud‐hosted learning model improved classification accuracy, achieving over 90% success across various lighting conditions. Dogan et al. applied ML‐enhanced smartphone platforms for water quality assessment, analyzing multiple ions using colorimetric strips.^[^ ^29^ ^]^ k‐NN, which showed the best performance among 23 classifiers, was integrated into a smartphone application and validated with real water samples. Similarly, Mostajabodavati et al. reported an LC‐based aptasensor for detecting *E. coli* in water samples.^[^ ^127^ ^]^ The sensor employed a textile grid‐anchored LC system that enabled homeotropic LC alignment without surface functionalization, reducing both development complexity and cost. The sensitivity of the sensor was complemented by the use of ML, such as ANN and XGBoost, to predict *E. coli* concentrations accurately, even in the presence of varying pH levels. The aptasensor exhibited a wide detection range (10–10^6^ CFU mL^−1^) and a LOD of 6 CFU mL^−1^, demonstrating high precision in environmental pathogen detection.

Another ML‐powered aptasensor was developed for the simultaneous detection of endocrine‐disrupting chemicals from the plastic industry, specifically di(2‐ethylhexyl) phthalate (DEHP) and bisphenol A (BPA), in river waters with varying pH levels (Figure 5).^[^ ^142^ ^]^ Using an Au nanoflower‐modified surface and integrated with differential pulse voltammetry, this aptasensor demonstrated exceptional sensitivity and specificity for both pollutants. The incorporation of the LSBoost algorithm effectively addresses the challenge of pH fluctuation, ensuring accurate pollutant detection in diverse environmental conditions. This technology greatly improves plastic pollution monitoring, providing a valuable tool for the environmental assessment and management of plastic contaminants in aquatic ecosystems. The development of more sophisticated ML algorithms and mobile integration makes these sensors even more accessible to non‐experts for monitoring water resources, ensuring a more sustainable approach to environmental conservation.

### 4.3 Food Safety and Agriculture

AI‐based biosensors enhance the detection of pathogens, antibiotic residues, and spoilage indicators, ensuring compliance with safety regulations and reducing foodborne risks. Most literature studies on AI‐based biosensors for food safety mainly focus on monitoring food freshness and spoilage. Guo et al. integrated cross‐reactive colorimetric barcodes with deep convolutional neural networks (DCNNs) to detect meat freshness in real‐time (Figure 5).^[^ ^143^ ^]^ The sensor utilized porous nanocomposites composed of chitosan, dye, and CA, which formed distinct scent fingerprints upon exposure to amine gases released by spoiling meat. A fully supervised DCNN, trained on 3475 labeled barcode images, enabled accurate classification of meat freshness with 98.5% accuracy, surpassing traditional Euclidean distance analysis (61.7%). The system was reported to be fast, nondestructive, and automated, allowing for real‐time monitoring via a smartphone application. In a similar approach, the MOF UiO‐66‐Br was incorporated into a colorimetric sensor array on an ice‐templated chitosan substrate, enabling the detection of ammonia, methylamine, and triethylamine released by spoiled shrimp, with a LOD of 37.17, 25.90, and 40.65 ppm, respectively.^[^ ^144^ ^]^ DCNNs were used to identify the scent fingerprint of shrimp freshness, achieving an accuracy of 99.94% with the Wide‐Slice Residual Network 50 (WISeR50) model. This integrated platform exhibited significantly better sensitivity compared to unmodified UiO‐66, providing a nondestructive, highly sensitive, and accurate solution for real‐time freshness monitoring. The same group of authors combined UiO‐66‐OH with polyvinyl alcohol, shifting their focus to chicken freshness monitoring. The LOD for trimethylamine was 80 ppm, and the system achieved 98.95% accuracy using the WISeR50 model.^[^ ^145^ ^]^ Building on previous advancements, another study combined a colorimetric sensor array (CSA) with bionic algorithms for the detection of total volatile basic nitrogen (TVB‐N) in beef.^[^ ^182^ ^]^ The CSA, with twelve color‐sensitive materials, captured scent information and generated scent fingerprints. Four optimization algorithms ‐ant colony optimization, particle swarm optimization, simulated annealing, and the whale optimization algorithm (WOA) ‐ were used to extract key variables, with WOA providing the best results. Based on two selected materials, a back‐propagation neural network model accurately determined TVB‐N with high precision. In a recent study, a CMS was developed for visual meat freshness monitoring using a CNN algorithm, addressing the limitations of traditional testing methods.^[^ ^153^ ^]^ The CMS, made from edible hydrogels containing pH‐responsive anthocyanins, changes color in response to the pH variations in meat tissue fluids, transitioning from pink to purple and eventually to dark blue as spoilage occurs. The sensor was combined with a DL‐assisted smartphone application, enabling accurate, portable, and real‐time freshness detection by classifying meat as “fresh,” “less fresh,” or “spoiled” with an accuracy of approximately 95.3%. Unlike previous studies, Dogan et al. embedded ML classifiers into a smartphone application for anthocyanin‐rich red cabbage extract‐based colorimetric monitoring of real‐time food spoilage.^[^ ^155^ ^]^ The AI‐integrated system utilized FG‐UV‐CD100 films that change color in response to ammonia from spoiled food. The best‐performing ML algorithm (RF), embedded into a smartphone application, achieved 98.8% classification accuracy and provided rapid, offline analysis within 0.1 s. Tested on real fish samples, it demonstrated 99.6% accuracy, offering a robust and easy‐to‐use tool for non‐experts in food spoilage detection and smart packaging. Similarly, a recent study utilized a smart pH‐responsive fluorescence film for food freshness detection.^[^ ^154^ ^]^ The film exhibited strong fluorescence across a wide pH range, and ML algorithms, including partial least squares and support vector machine regression (SVR), estimated food freshness indices with satisfactory accuracy. AI‐based biosensors are used not only for food freshness monitoring but also for quality assessment. A low‐cost fluorescence sensor combined with ML, including ANNs, was introduced, achieving 100% accuracy in classifying olive oil as extra virgin, virgin, or lampante.^[^ ^190^ ^]^ In contrast to traditional costly methods, it requires no dilution or pre‐processing, offering a simple and efficient alternative.

Following the emphasis on food freshness and quality, subsequent studies have shifted towards detecting antibiotic residues, which are among the most common contaminants in milk. For instance, a rapid and sensitive method was developed to detect residues of kanamycin (KAN), ampicillin (AMP), oxytetracycline (OTC), and sulfadimethoxine (SMX) in raw cow milk.^[^ ^147^ ^]^ The device integrated electro‐opto‐mechanic components to measure the variation in the absorption spectrum of milk, which was induced by the interaction between antibiotic‐specific aptamer‐modified AuNPs and antibiotic molecules. An SVM classifier was applied to analyze spectral data to determine the presence and concentration of antibiotics with high accuracy. With a low LOD of 0.25 times the maximum residue limit (MRL) and a linear range up to twice the MRL, this portable, high‐performance sensor enables on‐site antibiotic screening in farms and dairies, preventing contaminated milk from entering the food chain. Similarly, antibiotic detection in milk, reported by Zhou et al., was achieved using an optical immunosensor with functionalized polystyrene NPs as multiplex signal probes for one‐step detection of multiple antibiotics (chloramphenicol (CAP), KAN, neomycin (NEO)).^[^ ^148^ ^]^ Integrated with ML‐based transcoding analysis and a partial least squares regression classifier, the sensor enabled rapid (⩽30 min) and highly accurate (>99%) multiplexed detection across a broad concentration range (pg/mL to ng mL^−1^). Compared to standard chemiluminescence immunoassays, it offered improved accuracy, lower costs, and a simplified procedure for antibiotic quantification in milk. In another study, a bacterial array solid‐phase assay was developed for rapid antibiotic detection and classification using 15 genetically modified *E. coli* sensor strains with bioluminescence reporters.^[^ ^186^ ^]^ Exposure to 11 antibiotics triggered distinct luminescence patterns which were then analyzed via a Multiclass Decision Forest (MDF) model. The sensor identified and classified compounds in just three hours with 65% and 90% accuracy, respectively. Building on the trend of integrating advanced detection methods, a recent study introduced a novel approach that combined ML with dual SERS aptasensors to address the challenge of simultaneous detection of multiple food contaminants specifically CAP and estradiol (E2).^[^ ^185^ ^]^ Compared to other ML classifiers, ANN achieved superior precision, with R^2^ values ranging from 0.963 to 0.991 across various datasets. Signal probes were obtained using benzoic acid‐modified Au@AgNPs with SH‐CAP and SH‐E2 aptamers, while Fe_3_O_4_@Au nanoflowers integrated with complementary DNA of SH‐CAP and SH‐E2 aptamers served as capture probes. The applicability of the proposed approach was also demonstrated in sea bass samples.

Recently, AI‐based biosensors have also been applied to detect bacterial and mold contamination, demonstrating their increasing role in ensuring food safety and quality assurance. A portable immunosensor was developed for the early diagnosis of mastitis by detecting *S. aureus* in milk. Using a layer‐by‐layer chitosan‐CNT film with antibodies, the sensor achieved a LOD of 2.6 CFU mL^−1^.^[^ ^150^ ^]^ ML‐based DT enhanced data interpretation in complex milk samples, while electrical and EIS ensured high sensitivity and selectivity. Similarly, a quorum sensing‐based biosensor was used to identify ten bacterial species and their mixtures in water and milk.^[^ ^191^ ^]^ Peptides were crosslinked to microparticles and their interaction with bacteria led to aggregation on a μPAD. A smartphone‐linked fluorescence microscope was used to capture the images of particle aggregation. ML algorithms, including XGBoost, SVM, and k‐NN, classified bacteria with up to 91.67% accuracy in milk and 83.75% in water, enabling rapid and on‐site bacterial detection within 30 minutes. Apart from milk, detecting bacterial pathogens in various foods is crucial for food safety. Zhao et al. developed a fluorescence biosensor integrating machine vision and ML to detect viable *Salmonella typhimurium* without complex DNA extraction.^[^ ^149^ ^]^ Phages captured the bacteria, while Clostridium butyricum Argonaute facilitated target DNA cleavage, generating a fluorescence signal which was then analyzed via ML. This method demonstrated high sensitivity, with a LOD of 40.5 CFU mL^−1^, and was successfully validated across lettuce, pork, fish, chicken, milk, and orange juice. To advance AI‐assisted biosensing for quality deterioration, and mycotoxin detection, a whole‐cell biosensor array was developed for early mold detection in foodstuffs by identifying volatile markers from *Aspergillus flavus*‐infected peanuts.^[^ ^183^ ^]^ Using 14 *E. coli* stress‐responsive promoters and ML classifiers, particularly RF, the system achieved up to 100% accuracy in distinguishing healthy, moldy, and pre‐mold stages in peanuts and maize, offering an accurate and practical mold monitoring approach. In addition, a dual‐mode sensor utilizing V_2_C MXene nano‐enzyme materials was engineered for aflatoxin B1 (AFB_1_) detection.^[^ ^151^ ^]^ Fluorescence quenching resulting from the interaction between V_2_C MXene nanozyme and fluorescence‐labeled aptamers was disrupted by AFB_1_ binding, allowing recovery of fluorescence and thus highly sensitive dual‐mode detection. An ANN model was integrated into the sensor for automatic monitoring and analysis, resulting in LOD values of 0.0905 ng mL^−1^ and 0.6845 ng mL^−1^ for fluorescence and colorimetric detection, respectively.

Although AI‐based biosensors are less commonly applied in agriculture compared to healthcare, environmental, and food applications, they hold a significant potential to increase productivity, sustainability, and efficiency in farming. When integrated with biosensors, AI technologies such as ML, computer vision, and data analytics can optimize crop management, pest detection, irrigation, and soil health monitoring. By analyzing large amounts of data from sensors, drones, and satellites, AI could help farmers make informed decisions, reduce resource use, and increase crop yields. AI can also provide innovative solutions to global challenges such as food security, climate change, and environmental degradation by predicting the weather, detecting diseases early, and automating related processes.^[^ ^192^ ^]^ Recently, Parihar et al. reported the future applicability of an IoT‐integrated, smartphone‐assisted MXene‐enabled aptasensor for aflatoxin detection in food and agricultural products, emphasizing the importance of on‐site detection to prevent serious health risks associated with fresh fruit contamination.^[^ ^193^ ^]^ Similarly, an ultra‐portable electrochemical sensor was developed using ML to detect maleic hydrazide (MH), a sprout suppressant and plant growth inhibitor.^[^ ^152^ ^]^ ML algorithms, such as ANN and LS‐SVM, were integrated for intelligent and rapid analysis. According to the results, LS‐SVM outperformed ANN and exhibited a better R^2^ value than traditional linear analysis. The sensor successfully detected MH in sweet potato and carrot samples, offering higher sensitivity compared to traditional sensors.

## 5 Challenges, Opportunities, and Future Directions

As mentioned in Section 4, AI technology has led to significant advances in monitoring, diagnosis, and decision‐making by enhancing the capabilities of biosensors in healthcare, environmental monitoring, food safety, and agriculture.^[^ ^194^ ^]^ **Table** **3** provides a brief comparison of traditional biosensors with AI‐assisted biosensors. AI integration can significantly enhance the performance and various capabilities of biosensors including robustness, adaptability, sensitivity, specificity, data processing, personalization, calibration, and decision‐making. Despite these advancements, there are still several challenges associated with AI, particularly data‐related issues such as missing values, noisy signals, and imbalanced datasets, which can negatively impact model accuracy. Additionally, distribution shifts due to variations in sensor types, environmental conditions, and temporal changes can degrade the reliability of AI algorithms. The performance of AI is also influenced by the comprehensiveness and scope of the dataset. If the model is trained on a limited range of sensor data, it may struggle to generalize to new data distributions, causing unreliable predictions or poor performance. Moreover, limitations in biosensor capabilities, such as sensor precision, sampling rates, and data collection, can further impact performance. Besides, technological hurdles, such as biological variability, environmental factors, and complex data processing, along with ethical concerns like data privacy and algorithm transparency, add further complexity to the implementation process. Ensuring long‐term stability and adaptability across various applications also continue to be a major concern.^[^ ^195^, ^196^ ^]^ However, recent advancements in AI and biosensor technology present promising solutions. Innovations in sensor technologies with higher precision, better sampling rates, and more robust data collection methods, combined with AI algorithms designed to handle data variability, can enhance model generalization and reliability. Such developments are crucial for achieving more accurate and efficient biosensor applications across diverse environments.^[^ ^197^, ^198^, ^199^ ^]^ Future efforts will focus on improving sensor accuracy, enhancing model explainability, and addressing ethical and regulatory issues to ensure effective scalable solutions.^[^ ^200^ ^]^ Overcoming these obstacles is essential to fully realize the potential of AI‐enhanced biosensors in various applications. The following section will explore key technological hurdles, ethical considerations, and future directions.

**Table 3:** Comparison of traditional biosensors with AI‐assisted biosensors.

Feature	Conventional Biosensors	AI‐assisted Biosensors
Robustness/Adaptability	Limited adaptability to changes in environmental conditions	Dynamically learns and adapts to complex variations
Calibration	Manual and static calibration	Dynamic recalibration based on real‐time data
Data Processing	Relies on a calibration curve	Advanced data analysis involving pattern recognition Capable of complex and multiplexed biosensor data analysis
Error Handling	Manual	Automatic error detection and correction
Personalization	One‐size‐fits‐all	Suitable for personalized diagnostics as it can learn from individual profiles
Sensitivity	Dependent on and limited by hardware and design	Enhanced by AI‐based optimization and prediction
Complex Sample Analysis	Poor performance in noisy/complex samples	Effectively handles complex and noisy data
Decision Making Support	Requires expert interpretation	Capable of automatic decision support
Result Interpretability	Results are explainable	Not transparent and hard to interpret
Cybersecurity	Low risk	Vulnerable to software attacks and breaches

### 5.1 Technological Hurdles

Technological hurdles in AI‐driven biosensors involve complex challenges that require innovative solutions across various domains. One issue in AI is the interpretability of DL algorithms, which are often regarded as black‐box systems, making it difficult to fully understand their decision‐making processes.^[^ ^201^ ^]^ The compression of input data through neural network layers and the use of techniques like max‐pooling and dropout create challenges in explaining how specific outputs are derived from the internal workings of models. To overcome these issues, researchers are working towards interpretable algorithms that can provide insights into healthcare decision‐making. Another critical issue is the miniaturization of biosensors to create portable and wearable devices that maintain accuracy and durability. Furthermore, the challenge of detecting multiple biomarkers in complex biological fluids like saliva, sweat, and blood, while minimizing interference from proteins, urea, and minerals, requires the advancement of filtering technologies and data security measures.^[^ ^20^, ^202^, ^203^ ^]^ To address the challenge of detecting multiple biomarkers in complex biological fluids, ML algorithms are crucial in biosensor data analysis due to their ability to process large datasets, identify patterns, and differentiate between biomarkers while mitigating the effects of interference from other substances. Ensuring high sensitivity is also essential for accurate biomarker detection, and research efforts are focused on discovering new materials and functionalization methods to improve biosensor performance.^[^ ^204^ ^]^

Personalized healthcare solutions powered by AI require precise training frameworks to generate meaningful insights, enabling early disease detection and preventive care strategies.^[^ ^205^ ^]^ However, the adaptive learning ability of ML algorithms remains a critical challenge, as these algorithms must continuously adjust to new and evolving data inputs that can vary in type, quality, and complexity.^[^ ^206^ ^]^ This dynamic nature of the data requires the algorithms to effectively handle inconsistencies and noise while ensuring accurate predictions or classifications.^[^ ^207^ ^]^ As input from biological fluids can vary due to health conditions and environmental factors, the model must be robust and capable of real‐time processing to ensure consistent performance in various scenarios.^[^ ^208^ ^]^ The main challenge in developing AI algorithms for biosensors lies in the construction of data sets, where issues such as limited data volume, inconsistent data quality, and low accuracy of the algorithm create significant hurdles.^[^ ^209^ ^]^ Addressing the technological challenges in AI‐based biosensor technologies is essential to improve their robustness and ensure their effectiveness in real‐world applications. Moreover, there are some key issues associated with hardware including the need for more accurate and reliable sensors, and the development of more efficient AI‐based data processing methods.^[^ ^210^ ^]^ Overcoming these challenges is crucial to fully unlocking the potential of AI‐powered biosensors in clinical and field applications.

### 5.2 Ethical Considerations

As AI becomes increasingly integrated into biosensor technologies, addressing the ethical and security challenges associated with its application has become crucial to enable widespread implementation. Ensuring ethical considerations at every stage of the AI process, including data collection, pre‐processing, model training, evaluation, and deployment, is critical to prevent unfair decisions and biased outcomes.^[^ ^211^, ^212^ ^]^ Collected data should be anonymized to protect individual identities, ensuring privacy and ethical data handling throughout the AI development process. The selection of unbiased outcome labels and the implementation of rigorous pre‐analysis planning can further ensure that ML algorithms align with ethical objectives, such as transparency, privacy, and accountability.^[^ ^213^ ^]^ Security and data privacy are equally critical concerns, as biosensors collect sensitive information that could be vulnerable to unauthorized access and cyberattacks.^[^ ^214^ ^]^ To protect data integrity and confidentiality, robust encryption protocols and security measures must be implemented to prevent breaches and ensure privacy against external threats. Furthermore, comprehensive audits should be conducted to identify and address ethical risks systematically, considering the potential harms to specific demographic groups rather than generalized populations.^[^ ^215^ ^]^ By integrating ethical considerations, security measures, and inclusive design practices, the development of AI‐assisted biosensors can be guided toward responsible and equitable applications. Manipulation of AI‐integrated systems can cause misinterpretation of critical data and incorrect assessments of patient health status, food contamination levels, soil health, or environmental pollution, resulting in poor decision‐making and negative impacts on public health, crop yields, and ecosystem sustainability.^[^ ^216^ ^]^ To address such ethical issues, AI systems must be designed to be transparent and explainable. Transparency means that users should be able to understand how decisions are made by the system, while explainability ensures that those decisions can be justified and reviewed. Furthermore, accountability is essential, as developers and organizations using AI systems must be held responsible for the consequences of their actions.

### 5.3 Future Directions

Recent advancements in AI have attracted significant investments from countries and companies due to its potential to drive innovation, improve efficiency, and provide competitive advantages across various industries. These investments include increasing funding to construct new data centers and developing the large datasets essential for AI development. However, handling the massive volume of data generated today presents a significant challenge due to the limitations of current computing power. As the miniaturization of transistors in computer chips approaches its physical limit, the potential for further speed improvements using traditional transistor‐based technology is becoming increasingly constrained. Fortunately, with the advancement of quantum computing, new possibilities are emerging to overcome these limitations. Quantum computers leverage the principles of superposition and entanglement to perform complex calculations at unprecedented speeds, offering the potential to process vast amounts of data more efficiently and solve problems that are currently infeasible for classical computers. This capability has the potential to revolutionize AI by enabling faster processing of complex datasets and more sophisticated algorithms. As a result, quantum computing could significantly accelerate the development of AI algorithms, allowing for more accurate predictions, deeper insights, and the ability to solve problems that once seemed unsolvable, thus unlocking new possibilities for the future impact of AI across various sectors. One such area where these advances can have a profound impact is biosensor technology, which can provide real‐time, highly sensitive, and early detection of diseases such as cancer and neurological disorders. With the ability of AI to process complex medical data, quantum computing could drive faster drug discovery, and personalized medicine, and even support technologies like Neuralink, where AI algorithms analyze neural data for brain‐computer interface applications. These developments could potentially transform healthcare, enabling more precise treatments, earlier diagnosis, and novel solutions for conditions that were previously considered untreatable.^[^ ^217^ ^]^ The demand for such innovations is rapidly increasing, and many companies are now investing in similar research to explore the potential of this transformative technology.

In the near future, several key future directions will shape the evolution of AI‐powered biosensors, enhancing their capabilities, and accessibility.^[^ ^218^ ^]^ In healthcare applications, AI‐assisted biosensors will increasingly focus on real‐time health monitoring and predictive analytics, allowing proactive healthcare interventions. A practical example could be a wearable biosensor that continuously monitors lactate and hydration levels in athletes, monitoring physical state and providing personalized recommendations.^[^ ^219^ ^]^ The integration of biosensors with AI‐driven predictive models will accelerate drug discovery and clinical trials by providing real‐time patient response data. A biosensor patch used in clinical trials could monitor biomarkers related to drug efficacy and side effects, with AI analyzing trends to identify the most effective treatment protocols.^[^ ^220^ ^]^ To ensure widespread adoption, future developments will prioritize the creation of intuitive, user‐friendly interfaces. A smartphone application connected to a biosensor wristband could provide easy‐to‐understand health insights and personalized wellness recommendations based on AI analysis.^[^ ^221^ ^]^ In environmental monitoring, AI‐assisted biosensors are expected to contribute to early warning systems for environmental hazards such as oil spills and chemical leaks by providing real‐time detection of pollutants, heavy metals, and toxins. The integration of these sensors with the IoT and remote sensing will provide large‐scale monitoring of microplastics and nanoparticles in water and soil. As for agriculture, AI‐assisted biosensors are expected to support precision farming and optimize resource use in different areas, from soil health to crop disease monitoring. To increase food production efficiency, animal health monitoring, on‐site pesticide residue detection, pest and disease monitoring, etc. can be done instantly and accurately with AI‐assisted wearable biosensors. Likewise, AI‐assisted biosensors are expected to make significant contributions to processes such as rapid pathogen detection, smart packaging for real‐time spoilage monitoring, and food authenticity verification to prevent fraud in food safety applications. AI is also expected to be pivotal in improving multiplexed biosensor capabilities. Considering the complex nature of diseases, multiplexed biosensors have emerged as essential tools, especially in personalized therapy, to detect multiple biomarkers for holistic disease monitoring.^[^ ^222^ ^]^ The value of multiplexed sensing in environmental monitoring and food safety has also been stressed in various reviews.^[^ ^223^, ^224^, ^225^ ^]^ AI can address some prominent challenges in traditional multiplexed biosensors, such as signal interference, interpretation of large complex datasets, and signal drift. AI models can be trained to achieve accurate signal deconvolution, pattern recognition, and data interpretation, thereby differentiating overlapping signals for improved specificity and processing high‐dimensional datasets accurately in real time under varying conditions. Although signal drift is a common problem for biosensors, it might be a more challenging issue for multiplexed biosensors. AI models can actively learn from each analyte signal behavior and implement individual signal drift compensation techniques to maintain consistency and accuracy. In addition, the integration of biosensor data with blockchain technology will further increase traceability and security in food supply chains. However, several critical challenges must be addressed to fully unlock their capabilities. These include the need for more diverse and representative training datasets to mitigate algorithmic bias and improve generalization, as well as the creation of intuitive interfaces accessible to non‐experts. Addressing these issues will be crucial for advancing the widespread implementation and efficacy of these technologies in the aforementioned fields.^[^ ^226^ ^]^

AI has the potential to revolutionize biosensor technology not only with enhanced signal processing and performance but also by assisting in the discovery and rational design of novel sensing materials, thus reducing reliance on trial‐and‐error approaches. Material selection in biosensor development has a profound effect on performance and functionality. For example, adaptive and self‐healing materials such as self‐healing polymers, bio‐inspired materials, and dynamically responsive nanocomposites can significantly improve sensor durability and stability, ensuring long‐term reliability for continuous monitoring applications. Future research will focus more on developing new AI algorithms and methods involving physical models and simulations for designing novel sensing materials. In that context, AI will be used to screen existing databases of materials to design and optimize the general structure, geometric parameters, surface chemistry, functionality, and performance of such materials for biosensor applications.^[^ ^227^, ^228^, ^229^ ^]^ The use of such an approach will also assist researchers in tuning bioreceptor compatibility, electrical conductivity, and molecular selectivity of nanomaterials such as functionalized CNTs, graphene derivatives, or MOFs. The integration of AI in material synthesis will also promote sustainable biosensing materials, such as biodegradable nanomaterials and eco‐friendly polymers, reducing environmental impact. AI‐driven microfabrication, 3D/4D printing, and flexible electronics will significantly contribute to the development of ultra‐sensitive, wearable, and implantable sensors. Furthermore, AI‐supported molecular dynamics simulations and quantum chemical calculations will be a useful tool in the rational design and development of high‐performance biosensors by investigating sensing material and target molecule interactions at the atomic scale.^[^ ^230^, ^231^ ^]^

Due to ethical concerns and data security, future research will prioritize secure AI frameworks that preserve privacy.^[^ ^232^ ^]^ Governments are increasingly enacting laws and regulations to address ethical issues in AI, and this trend is gaining momentum worldwide as more nations recognize the importance of ensuring responsible and fair AI development.^[^ ^233^ ^]^ In conclusion, the integration of AI with biosensor technologies offers significant potential to transform healthcare, environmental monitoring, agriculture, and food safety. However, overcoming the current technological hurdles, addressing ethical considerations, and filling research gaps are essential to fully realizing the capabilities of AI in biosensing. Prioritizing these aspects will enable the development of more precise, effective, and ethically sound AI‐powered biosensors in the near future.

## Conflict of Interest

The authors declare no conflict of interest.
